# The NEDD4‐1 E3 ubiquitin ligase: A potential molecular target for bortezomib sensitivity in multiple myeloma

**DOI:** 10.1002/ijc.32615

**Published:** 2019-08-24

**Authors:** Xi Huang, Huiyao Gu, Enfan Zhang, Qingxiao Chen, Wen Cao, Haimeng Yan, Jing Chen, Li Yang, Ning Lv, Jingsong He, Qing Yi, Zhen Cai

**Affiliations:** ^1^ Bone Marrow Transplantation Center, Department of Hematology The First Affiliated Hospital, School of Medicine, Zhejiang University Hangzhou China; ^2^ Department of Pharmacy The First Affiliated Hospital, School of Medicine, Zhejiang University Hangzhou China; ^3^ Center for Hematologic Malignancy Research Institute, Houston Methodist Houston TX; ^4^ Institute of Hematology, Zhejiang University China

**Keywords:** NEDD4‐1, Akt, Bortezomib, Ubiquitination, Multiple myeloma

## Abstract

E3 ubiquitin ligases primarily determine the substrate specificity of the ubiquitin‐proteasome system and play an essential role in the resistance to bortezomib in multiple myeloma (MM). Neural precursor cell‐expressed developmentally downregulated gene 4‐1 (NEDD4‐1, also known as NEDD4) is a founding member of the NEDD4 family of E3 ligases and is involved in the proliferation, migration, invasion and drug sensitivity of cancer cells. In the present study, we investigated the role of NEDD4‐1 in MM cells and explored its underlying mechanism. Clinically, low NEDD4‐1 expression has been linked to poor prognosis in patients with MM. Functionally, NEDD4‐1 knockdown (KD) resulted in bortezomib resistance in MM cells *in vitro* and *in vivo*. The overexpression (OE) of NEDD4‐1, but not an enzyme‐dead NEDD4‐1‐C867S mutant, had the opposite effect. Furthermore, the overexpression of NEDD4‐1 in NEDD4‐1 KD cells resensitized the cells to bortezomib in an add‐back rescue experiment. Mechanistically, pAkt‐Ser473 levels and Akt signaling were elevated and decreased by NEDD4‐1 KD and OE, respectively. NEDD4‐1 ubiquitinated Akt and targeted pAkt‐Ser473 for proteasomal degradation. More importantly, the NEDD4‐1 KD‐induced upregulation of Akt expression sensitized MM cells to growth inhibition after treatment with an Akt inhibitor. Collectively, our results suggest that high NEDD4‐1 levels may be a potential new therapeutic target in MM.

## Abstract 2

**What's new?**

While multiple myeloma (MM) remains an incurable disease, therapeutic advances have greatly improved its clinical management. Among the agents most effective against MM is the proteasome inhibitor bortezomib, though many patients eventually develop drug resistance. Here, decreased expression of the tumor suppressor NEDD4‐1 was found to serve a key role in modulating MM sensitivity to bortezomib. Reduced NEDD4‐1 expression was associated with increased PTEN/PI3K/Akt signaling and tumor growth. Experiments showed that NEDD4‐1 normally binds directly to Akt, enhancing its ubiquitination and thereby exerting anti‐MM effects. The data warrant further investigation of NEDD4‐1 reactivation as a novel therapeutic strategy for MM.

**Abbreviations**
Afu: afuresertib
BM: bone marrow
Bor: bortezomib
CCK‐8: cell counting kit‐8
CHX: cycloheximide
ELISA: enzyme‐linked immunosorbent assay
HCQ: hydroxychloroquine sulfate
HMCLs: human myeloma cell lines
I3C: indole‐3‐carbinol
KD: knockdown
MGUS: monoclonal gammopathy of undetermined significance
MM: multiple myeloma
MOI: multiplicity of infection
NOD‐SCID: nonobese diabetic‐severe combined immunodeficient
OE: overexpression
PBS: phosphate‐buffered saline
PI: propidium iodide
qRT‐PCR: quantitative real‐time polymerase chain reaction
RIPA: radioimmunoprecipitation assay
SDS‐PAGE: sodium dodecyl sulfate‐polyacrylamide gel electrophoresis
TBS‐T: tris‐buffered saline with Tween

## Introduction

Multiple myeloma (MM) is a hematological malignancy characterized by the monoclonal expansion of plasma cells in the bone marrow (BM) and/or extramedullary sites and is typically accompanied by the secretion of monoclonal immunoglobulins.^1^ MM is the second most frequent hematological malignancy, with an age‐adjusted incidence of 6.7 per 100,000 per year based on cases from 2011 to 2015. The 5‐year survival rate for patients with MM is 50.7% based on data from 2008 to 2014.^2^ During the last few years, various approaches, such as the proteasome inhibitor bortezomib (Bor), the immunomodulatory drug lenalidomide, monoclonal antibodies and autologous stem cell transplantation have been used in MM therapy. Bor is one of the most effective drugs for the treatment of MM, but most MM patients develop drug resistance, which results in recurrence and eventually a poor prognosis.^3^ A better understanding of the genetic make‐up of Bor‐resistant myeloma cells is urgently needed for the development of novel agents to treat MM patients.^4^

To some extent, Bor resistance is attributed to abnormalities in the ubiquitin‐proteasome pathway, such as variations in the structure of the proteasome and its associated enzymes.^5^, ^6^, ^7^ The ubiquitin‐proteasome pathway is the main method for degrading intracellular protein and plays an important role in tumor development and localization.^8^ In eukaryotes, ubiquitination involves three classes of enzymes: ubiquitin‐activating enzyme (E1), ubiquitin‐binding enzyme (E2) and ubiquitin ligase (E3), and E3s determine substrate specificity. In the three‐step enzymatic cascade reaction, proteins bind to ubiquitin (Ub) and are selectively labeled, and then they are degraded by the 26S proteasome.^9^ Ub consists of seven lysine residues (K6, K11, K27, K29, K33, K48 and K63), one C‐terminal glycine (Gly) site and an N‐terminal methionine (Met1). The K48‐linkage is generally considered to be proteasome pathway‐related ubiquitination, while the K63‐linkage is involved in DNA repair, protein trafficking, autophagy, immunity, inflammation and other processes.^10^ E3s are key regulators that determine the spatiotemporal regulation of proteins. Neuronal precursor cell‐expressed developmentally downregulated gene 4‐1 (NEDD4‐1), which was initially discovered from mouse neuronal precursor cells as a regulator of neuronal function,^11^ is a critical member of the NEDD4 family and a HECT domain‐containing ubiquitin E3 ligase. The NEDD4 family consists of nine members, including NEDD4‐1, NEDD4‐2, Smurf2, WWP2 and Itch, and is highly evolutionarily conserved. NEDD4 family members have a common domain architecture with a C‐terminal HECT domain that transfers Ub, an N‐terminal C2 domain that locates the protein and WW domains that identify and bind the PPXY motifs of substrates.^12^, ^13^ As an E3, NEDD4‐1 has been reported to ubiquitinate many substrates, including N‐Myc, C‐Myc and RAS, and exert a tumor‐suppressing effect.^14^, ^15^ Extensive studies have shown that NEDD4‐1 plays an essential role in the proliferation, migration and invasion of multiple malignancies, including nonsmall‐cell lung cancer,^16^ colorectal cancer^17^, ^18^ and breast cancer,^19^, ^20^, ^21^, ^22^ and in their sensitivities to anticancer therapies. However, the functional significance of NEDD4‐1 in MM remains unknown.

Akt, a highly evolutionarily conserved serine‐threonine kinase, is ubiquitous in MM, and high Akt expression is especially correlated with the recurrence of refractory disease.^23^ PTEN/PI3K/Akt signaling, including phosphatidylinositol‐3 kinase (PI3K), its downstream kinase Akt, and the negative regulator PTEN, is frequently altered in tumors.^24^, ^25^ As an important PTEN/PI3K/Akt signaling hub, Akt regulates cell metabolism, cell cycle progression, proliferation and differentiation by regulating more than 100 downstream target substrates.^26^ Recent studies have reported that steady levels of Akt can also be ubiquitinated. The RING finger family E3 ligase TRAF6 ubiquitinates Akt and strengthens Akt membrane recruitment and phosphorylation, which are dependent on growth factor stimulation.^27^ Furthermore, NEDD4‐1 is an E3 ligase targeting Akt for phosphorylation and nuclear trafficking in the IGF‐I response.^28^, ^29^ Therefore, we evaluated how Akt can be regulated by NEDD4‐1 E3s in MM and whether it plays a role in Bor sensitivity.

In this report, we identified NEDD4‐1 as an E3 ligase of Akt in MM. NEDD4‐1 knockdown (KD) resulted in decreased Bor sensitivity, elevated pAkt‐Ser473 levels and decreased Akt ubiquitination. NEDD4‐1 overexpression (OE) induced increased Bor sensitivity and led to increased pAkt‐Ser473 proteasomal degradation and the deactivation of the PTEN/PI3K/Akt signaling. The inhibition of NEDD4‐1 expression promoted tumor growth, and NEDD4‐1 OE exerted an antitumor effect *in vivo*. Together, these data suggest that NEDD4‐1 may be a promising drug target in MM.

## Materials and Methods

### HMCLs, BM samples, drugs and antibodies

The human myeloma cell lines (HMCLs) RPMI8226, MM.1S and the hepatocellular carcinoma cell line Hep3B were purchased from the Cell Bank of the Chinese Academy of Science; NCI‐H929, ARP‐1, LP‐1, CAG, ARK and JJN3 cells were kindly provided by Dr. Qing Yi (Center of Hematologic Malignancy Research Institute, Houston Methodist, Houston, TX). All human cell lines have been authenticated using STR profiling and all experiments were performed with mycoplasma‐free cells. BM samples from MM patients and peripheral blood mononuclear cells (PBMCs) from healthy donors were obtained after informed consent was provided following approval by the Ethics Committee of the First Affiliated Hospital, College of Medicine, Zhejiang University. CD138^+^ cells were purified by positive selection using CD138 microbeads (Miltenyi Biotech, Auburn, CA). Bor, melphalan (Mel), Q‐VD‐Oph, hydroxychloroquine sulfate (HCQ) and NQDI‐1 were obtained from Selleck Chemicals LLC (Houston, TX). Afuresertib (Afu), Bax inhibitor peptide V5 (Baxi), cycloheximide (CHX) and MG‐132 were obtained from MedChemExpress (Monmouth Junction, NJ). IGF‐I was purchased from Novus Biologicals, LLC (Littleton, CO). Dexamethasone and dimethyl sulfoxide (DMSO) were purchased from Sigma (St. Louis, MO). The FITC Annexin V Apoptosis Detection Kit, APC Annexin V and Propidium Iodide (PI) Staining Solution were obtained from BD (PharMingen, San Diego, CA). Primary antibodies against NEDD4‐1, PI3K‐β, Akt, pAkt‐Ser473 and ubiquitin were procured from Abcam (Cambridge, UK). GAPDH, Lamin B1, cleaved Caspase‐3, PARP‐1, PTEN, Bcl‐2, P21, P27, Ubiquitin, k48‐linkage polyubiquitin and k63‐linkage polyubiquitin were purchased from Cell Signaling Technology (Danvers, MA). HA was obtained from Roche (Basel, Switzerland). IgG, GST and His were obtained from GenScript (Piscataway, NJ). Horseradish peroxidase (HRP)‐conjugated antirabbit and antimouse antibodies were obtained from Jackson ImmunoResearch Laboratories (Lancaster, PA).

### Cell culture and transfection

The HMCLs and Hep3B were cultured in RPMI‐1640 medium or DMEM containing l‐glutamine (Corning Cellgro, Corning, NY) supplemented with 10% fetal bovine serum (Thermo Fisher Scientific, Gibco, Waltham, MA) at 37°C in a humidified atmosphere with 5% CO_2_. For stable NEDD4‐1 KD, HMCLs were transfected with green fluorescent protein‐containing (GFP) shRNA lentiviral particles directed against human NEDD4‐1 or with the shScramble vector. Cells were selected in culture media containing puromycin (2 μg/ml) for 2 weeks. HA‐NEDD4‐1 plasmid‐packaged (generously obtained from Yi Sun^30^) lentiviral particles, HA‐NEDD4‐1‐C867S (an enzyme‐dead HECT domain mutant) and EV‐transfected controls were transfected into MM cells, which were selected with puromycin as described above (Genechem Co., Ltd., Shanghai, China). For transient expression, the NEDD4‐1 adenovirus and the control vector were transfected into MM cells with an appropriate multiplicity of infection (MOI; Genechem Co., Ltd.). The sequences of Akt siRNA and nonsilencing control siRNA were 5′‐GCACCUUCAUUGGCUACAA‐3′ and 5′‐UUCUCCGAACGUGUCACGU‐3′, respectively.

### Cell proliferation assay

A cell counting kit‐8 (CCK‐8) assay (Dojindo, Japan) was used to measure MM cell proliferation and viability. MM cells (5 × 10^3^/100 μl/well) were seeded in 96‐well plates and treated with the indicated concentrations of drugs at 37°C in a humidified atmosphere with 5% CO_2_. After incubation for the indicated times, the cells were treated with 10 μl of CCK‐8 solution and incubated at 37°C for another 2 hr. Then, absorbance was measured at 450 nm using a microplate reader (Bio‐Rad, Model 680). Cell viability (%) = OD value of the test sample/OD value of the control × 100.

### Cell apoptosis assay and cell cycle analysis

To examine apoptotic cells, 1 × 10^5^/ml MM cells were plated in 24‐well plates with corresponding treatments. Then, the cells were harvested, washed twice with phosphate‐buffered saline (PBS), resuspended in 200–300 μl of staining buffer, and stained with Annexin V FITC/APC and PI according to the manufacturer's instructions. After incubating for 15 min at room temperature, the cells were detected using flow cytometry (BD Biosciences, USA), and the data were analyzed using FlowJo X 10.0.7. For cell cycle analysis, HMCLs were cultured at 3 × 10^5^/3 ml/well with or without Bor at a constant ratio in 12‐well plates for 24 hr. Then, the cells were harvested and washed twice with PBS and permeabilized with precooled 75% ethanol at 4°C overnight. The next day, the cells were washed with PBS and treated with 0.01% RNase A for 30 min at 37°C. The cells were then incubated with 0.5% PI away from light for 30 min. The DNA content was detected by flow cytometry, and the data were analyzed with ModFit software (version 3.2, Verity Software House).

### Quantitative RT‐PCR

Total RNA was isolated from the indicated cells using TRIzol (Invitrogen, Waltham, MA) according to the manufacturer's instructions. cDNA was synthesized using the PrimeScript™ RT Reagent Kit with gDNA Eraser (Takara, Japan). qRT‐PCR was performed with iTaq Universal SYBR Green Supermix (Bio‐Rad, Hercules, CA) and the Bio‐Rad CFX96 real‐time system on an iCycler (Bio‐Rad). Quantification was based on ΔΔCT calculations and normalized to the expression of GAPDH as the loading control. Each sample was run in triplicate. The primers used for qRT‐PCR were as follows: NEDD4‐1 (forward 5′‐TCCAATGATCTAGGGCCTTTACC‐3′; reverse 5′‐TCCAATGATCTAGGGCCTTTACC‐3′) and GAPDH (forward 5′‐ACGGATTTGGTCGTATTGGGC‐3′; reverse 5′‐ACGGATTTGGTCGTATTGGGC‐3′).

### Western blotting analysis

To evaluate the changes in cellular protein levels, MM cell lines were harvested and washed twice with PBS, and total protein was extracted with lysis buffer containing a mixture of protease and phosphatase inhibitors (Thermo Fisher Scientific). The suspension was incubated for 30–60 min at 4°C and then centrifuged at 16,000 rpm for 20 min at 4°C. The supernatants containing total cellular protein were collected for Western blotting. The samples were boiled at 100°C for 10 min after mixing with 4× sodium dodecyl sulfate (SDS) loading buffer (Invitrogen). The proteins (20–40 μg) were separated by 4–20% SDS‐polyacrylamide gel electrophoresis (PAGE) and transferred to polyvinylidene difluoride membranes (Merck Millipore, Germany). After blocking with 5% nonfat milk or 5% bovine serum albumin for 2 hr, the membranes were incubated with specific primary antibodies overnight at 4°C. The next day, the membranes were washed with Tris‐buffered saline with Tween (TBS‐T) three times and incubated with HRP‐conjugated antirabbit or antimouse antibodies at room temperature for 1 h. The membranes were then washed with TBS‐T three times, and the signals were detected using the ChemiDoc MP Imaging System (Bio‐Rad) with an enhanced chemiluminescence detection kit (Biological Industries Israel Beit Haemek Ltd., Israel). Protein levels were analyzed with Image Lab software (Bio‐Rad, USA), and results were shown in Supporting Information Figures S7–S9 for details.

### Co‐IP, *in vivo* ubiquitination and GST pulldown assays

To immunoprecipitate endogenous and exogenous proteins, whole‐cell extracts were precleared with protein A and G beads (Life Technologies), followed by overnight incubation at 4°C with IgG and other relevant antibodies. The beads were washed three times with lysis buffer, and the immunoprecipitation complexes were subjected to SDS‐PAGE. The Dynabeads® Coimmunoprecipitation Kit was purchased from Thermo Fisher Scientific Inc. To detect Akt and pAkt ubiquitination, NEDD4‐1‐KD or NEDD4‐1‐OE cell lysates were lysed in RIPA buffer with an additional 1% SDS and heated at 120°C for 5 min to dissociate the protein complexes. The heated lysates were diluted in a 10× volume of RIPA buffer. Akt, pAkt or Ub was immunoprecipitated from the cell lysates after incubation of the antibodies with Dynabeads and then blotting with antibodies. To verify the direct binding of NEDD4‐1 to Akt by pulldown, GST‐NEDD4‐1 and His‐Akt were purified from *Escherichia coli*. GST‐beads or His‐beads were used for incubation with the purified NEDD4‐1 or Akt at 4°C for 2 hr with rotation. The beads were washed 5 times with PBS. Then, the Akt or NEDD4‐1 protein lysate obtained in the above step was added, and binding was performed by rotation overnight at 4°C. The beads were washed five times with PBS, and the supernatants were obtained by centrifugation for SDS‐PAGE.

### ELISA

We collected approximately 0.7 ml of peripheral blood from the eyeballs of mice. The blood was incubated in a 37°C water bath for 10 min, placed at 4°C for 15 min, and centrifuged at 5,000 rpm/min for 20 min. This treatment yielded nearly 0.15 ml of supernatant. The levels of soluble lambda light chain in the peripheral blood supernatants harvested from ARP‐1 xenograft NOD/SCID mice were measured using the Human Lambda enzyme‐linked immunosorbent assay (ELISA) Kit (Bethyl Laboratories, Montgomery, TX) according to the manufacturer's instructions. The sensitivity of the kit was 1.37 ng/ml.

### *In vivo* xenograft studies

Three‐week‐old male NOD‐SCID (nonobese diabetic‐severe combined immunodeficient) mice were purchased from Vital River Laboratory Animal Technology Co. Ltd. (Beijing, China) and housed in the animal facility of Zhejiang University School of Medicine. After 1 week of acclimatization, the NOD‐SCID mice were injected subcutaneously into the left flanks with 5 × 10^6^ ARP‐1 cells resuspended in 50 μl of RPMI‐1640. After approximately 11 days, when the established tumors reached approximately 100–130 mm^3^, the mice were randomly divided into eight groups and then received intraperitoneal injections of PBS or Bor (0.5 mg/kg, every 3–4 days). Tumor diameters were measured with calipers when Bor or PBS was injected, and the tumor volume was calculated as 4π/3 × (*a*/2)^2^ × *b*/2, where *a* is the tumor width and *b* is the tumor length. The mice were sacrificed when the tumor volumes reached approximately 3,000 mm^3^. All animal experiments were carried out in accordance with the procedures and protocols of the Animal Ethics Committee of the First Affiliated Hospital, College of Medicine, Zhejiang University.

### Immunofluorescence and immunohistochemistry analyses

Paraformaldehyde‐fixed, Triton X‐100‐permeabilized cells from BM biopsy tissues from MM patients as well as HMCLs were used for immunofluorescence staining to analyze the expression and localization of NEDD4‐1 in CD138^+^ MM cells and the relationship between NEDD4‐1 and pAkt‐Ser473. Additionally, paraformaldehyde‐fixed, paraffin‐embedded sections (5 μm) of tumor tissues from tumor‐bearing NOD‐SCID mice were used for immunohistochemical staining to analyze NEDD4‐1, Akt, pAkt, Ki67, cleaved Caspase‐3, cleaved PARP‐1, P21 and PTEN expression. The data were analyzed using Quant center, Pannoramic viewer (3D HISTECH, Hungary) and Image‐pro plus 6.0 (Media Cybernetics, Inc., Rockville, MD). Average optical (AO) = IOD/AREA.

### Database

**Figure 1:**
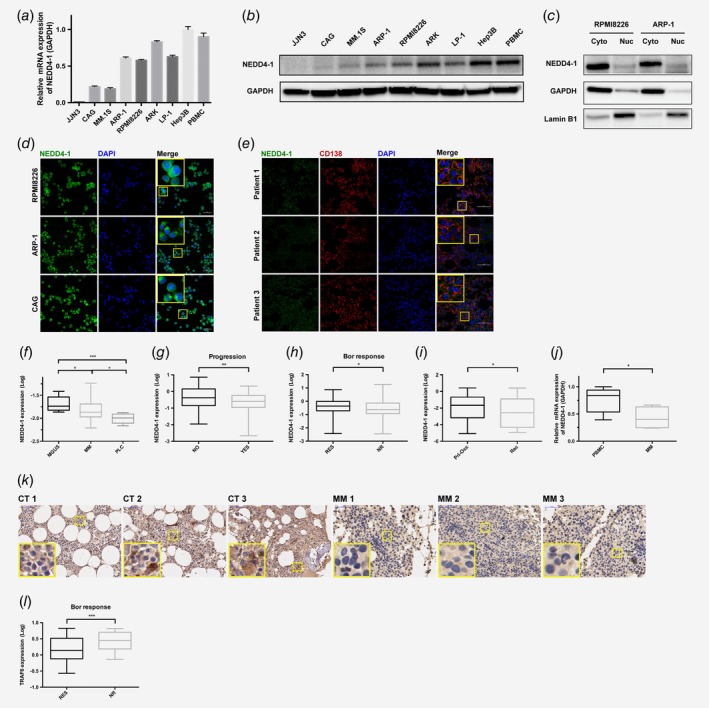
Clinical significance of NEDD4‐1 in MM. (*a*, *b*) RT‐PCR and Western blotting analysis of HMCL lysates showed the levels of NEDD4‐1 mRNA and protein expression. The cell lysates were prepared from the indicated cell lines, followed by Western blotting with an equal amount of protein loaded and detection using the indicated antibodies. (*c*–*e*) Western blotting and immunofluorescence staining (phase‐contrast microscopy and confocal microscopy) analysis of the subcellular localization of NEDD4‐1 in HMCLs and CD138^+^ cells from the BM biopsy tissues of MM patients. Cyto refers to the cytoplasm, and Nuc refers to the nucleus. Nuclei were stained with DAPI. Scale bars, 50 μm. (*f*) NEDD4‐1 expression in the purified plasma cells of patients with monoclonal gammopathy of undetermined significance (MGUS, *n* = 8), multiple myeloma (MM, *n* = 133) and plasma cell leukemia (PCL, *n* = 8). The *y*‐axis indicates the median log value of NEDD4‐1 retrieved from the original dataset. (*g*, *h*) NEDD4‐1 expression according to progression (NO, *n* = 52; YES, *n* = 174) and the Bor response (RES, *n* = 75; NR, *n* = 82). (*i*) NEDD4‐1 expression in patients with primary occurrence (Pri‐Occ, *n* = 81) and with recurrence (Rec, *n* = 37). (*j*) RT‐PCR analysis of the mRNA level of NEDD4‐1 in peripheral blood mononuclear cells (PBMCs, *n* = 11) and CD138^+^ cells from primary MM cells (MM, *n* = 11). (*k*) BM biopsies from healthy donors (CTR) or MM patients (MM) were analyzed by immunohistochemical staining for NEDD4‐1 (brown staining). Three individual samples are shown. (*l*) TRAF6 expression according to the Bor response (RES, *n* = 75; NR, *n* = 82). Western Blot bands are derived from separate experiments but only one representative loading control is shown. *p < 0.05, **p < 0.01, ***p < 0.001. [Color figure can be viewed at http://wileyonlinelibrary.com]

We specifically analyzed the log_2_‐transformed median‐centered values of NEDD4‐1 genes in the Oncomine database from Agnelli Myeloma 3 Statistics (comparison of the gene expression of purified CD138^+^ BM plasma cells from monoclonal gammopathy of undetermined significance (MGUS), MM and plasma cell leukemia (PCL) patients), Mulligan Myeloma Statistics (assessment of the feasibility of prospective pharmacogenomics research in multicenter international clinical trials of Bor in MM) and Burington Myeloma Statistics (comparison of the gene expression in BM plasma cells after short‐term *in vivo* exposure to single‐agent chemotherapeutics). The differential NEDD4‐1 expression among MGUS, MM and PCL patients, patients with different disease statuses, and patients with differential outcomes was evaluated using one‐way ANOVA or unpaired *t*‐test, as stated in the legends of Figure 1, using Prism software (GraphPad).

### Statistical analysis

All data are presented as the mean ± standard deviation (SD). All analyses were conducted using GraphPad Prism 6.0 (GraphPad Software, San Diego, CA). Two‐tailed Student's *t*‐test was used to estimate significant differences between two groups. All *p* values <0.05 were recognized as statistically significant. All experiments were performed in triplicate as three independent assays (*/#/&*p* < 0.05, **/##/&&*p* < 0.01, ***/###/&&&*p* < 0.001).

## Results

### Clinical significance of NEDD4‐1 expression in MM

The expression and localization of NEDD4‐1 were detected in HMCLs and primary MM cells. Various amounts of NEDD4‐1 were found in seven MM cell lines, Hep3B and PBMCs. Hep3B and PBMCs had relatively high expression levels of NEDD4‐1 mRNA and protein, but JJN3 had relatively low expression levels (Figs. 1 *a* and 1*b*). Western blotting and immunofluorescence staining showed that NEDD4‐1 was localized to both the cytoplasm and nucleus but was primarily found in the cytoplasm of HMCLs and CD138^+^ cells from the BM biopsy tissues of MM patients (Figs. 1 *c–*1*e* and Supporting Information Fig. S1*a*).

To evaluate whether the expression of NEDD4‐1 was associated with the development of MM and the compatibility of targeting NEDD4‐1 for MM treatment, we downloaded three independent MM‐related microarray datasets from the publicly available gene expression profiling datasets of Oncomine (available online). Agnelli Myeloma 3 Statistics revealed that the expression of NEDD4‐1 was higher in eight subjects with MGUS and 133 subjects with MM than in eight subjects with PCL (Fig. 1 *f*), suggesting that NEDD4‐1 expression was inversely correlated with aggressiveness. Using Mulligan Myeloma Statistics, we found that elevated NEDD4‐1 expression was a prognostic indicator for nonprogression and a Bor response in MM patients (Figs. 1 *g* and 1*h*). In addition, we found that TRAF6 was positively correlated with Bor resistance (Fig. 1 *l*). According to the Burington Myeloma Statistics, patients with recurrence exhibited lower NEDD4‐1 expression levels than those with primary occurrence (Fig. 1 *i*). Furthermore, PBMCs from 11 healthy donors expressed higher NEDD4‐1 levels than CD138^+^ cells from 11 MM patients (Fig. 1 *j*). The immunohistochemistry analysis of MM and healthy BM showed that MM BM had decreased NEDD4‐1 expression (Fig. 1 *k*). Overall, these data analyses strongly suggested that low NEDD4‐1 expression in malignant plasma cells is a risk factor in MM.

### NEDD4‐1 mediates the Bor resistance of MM cells

**Figure 2:**
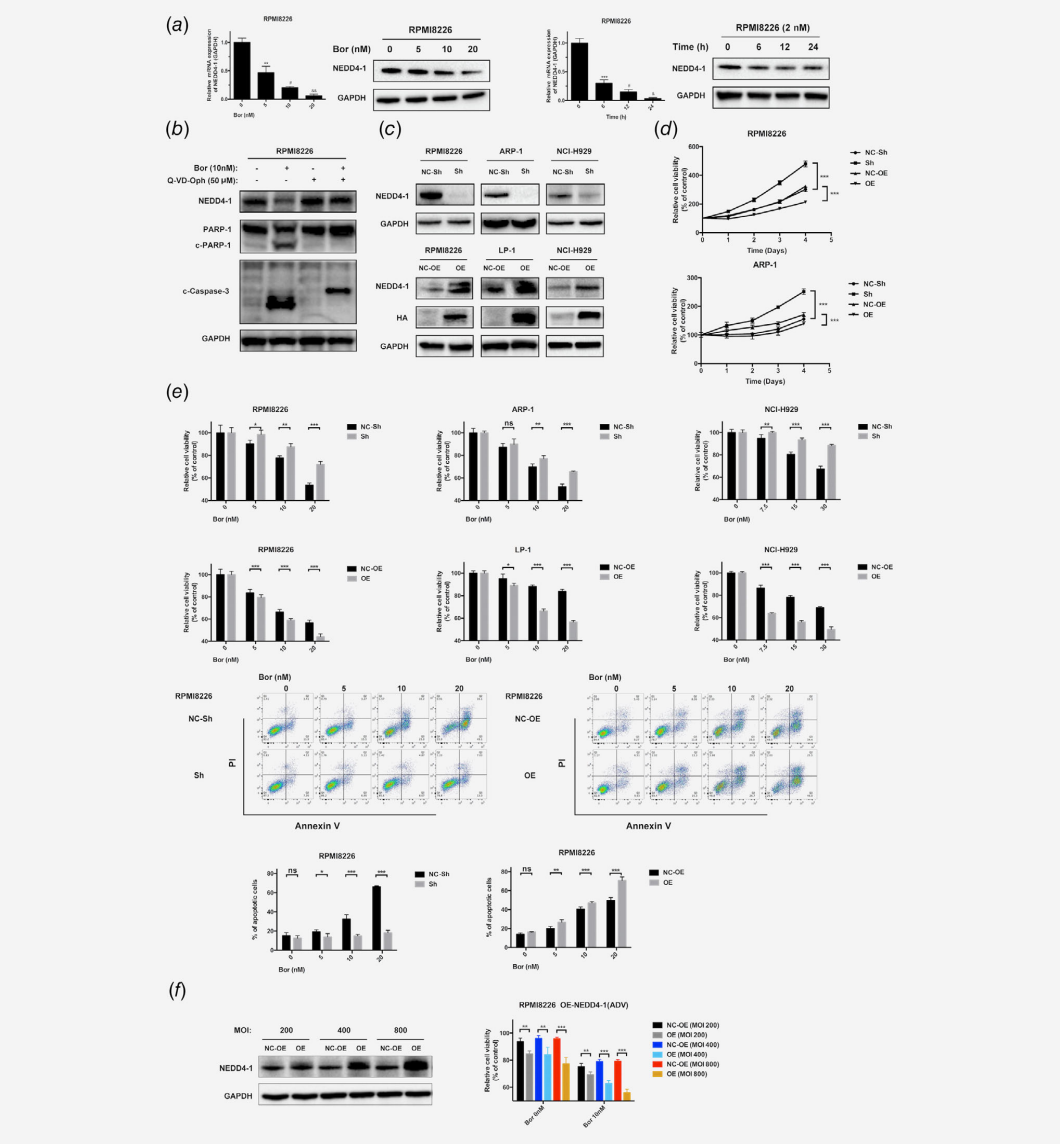
NEDD4‐1 mediates Bor resistance in MM cells. (*a*) RPMI8226 cells were treated with different concentrations of Bor (0, 5, 10 and 20 nM) for 24 hr or 2 nM Bor for different durations (0, 6, 12 and 24 hr). The mRNA and protein levels of NEDD4‐1 were evaluated by RT‐PCR and Western blotting. (*b*) RPMI8226 cells were treated with or without Bor (10 nM) or Q‐VD‐Oph (50 mM). Whole‐cell extracts were analyzed by Western blotting with NEDD4‐1, PARP‐1, c‐Caspase and GAPDH antibodies. (*c*) Verification of the effect of NEDD4‐1 knockdown (KD) or overexpression (OE) in HMCLs. The protein levels of NEDD4‐1 were evaluated by Western blotting. (*d*) NEDD4‐1 KD or OE HMCLs were seeded in 96‐well plates with a certain concentration of Bor. After incubation for different durations at 37°C, cell proliferation was measured by CCK‐8 assay. (*e*) NEDD4‐1 KD and OE HMCLs were treated with the indicated concentration of Bor. After 24 hr of incubation at 37°C, cell viability was measured by CCK‐8 assay, and cell apoptosis was detected by flow cytometry. Annexin V‐positive cells were considered apoptotic cells. The right histograms show the percentage of cells undergoing apoptosis. “ns” refers to “nonsignificance”. (*f*) RPMI8226 cells were transfected with an increasing MOI of NEDD4‐1 adenovirus. Whole‐cell extracts were analyzed by Western blotting with NEDD4‐1 antibodies. RPMI8226 cells expressing different amounts of NEDD4‐1 or controls were seeded in 96‐well plates with the indicated concentration of Bor, and after 24 hr of incubation at 37°C, cell viability was measured by CCK‐8 assay. Western Blot bands are derived from separate experiments but only one representative loading control is shown (*/#/&*p* < 0.05, **/##/&&*p* < 0.01, ***/###/&&&*p* < 0.001). [Color figure can be viewed at http://wileyonlinelibrary.com]

To investigate the relationship between NEDD4‐1 and the vulnerability to Bor in MM, the NEDD4‐1 level in Bor‐treated HMCLs was measured in RPMI8226 and ARP‐1 cells. Western blotting and RT‐PCR revealed that in MM cells, Bor treatment was associated with a reduction in NEDD4‐1 in a dose/time‐dependent manner, as shown in Figure 2 *a* and Supporting Information Figure S1*b*. Furthermore, Bor treatment also reduced the expression of NEDD4‐1 in the nucleus (Supporting Information Fig. S1*c*). However, the level of NEDD4‐1 did not decrease when HMCLs were incubated with Bor in the presence of pan‐caspase inhibitors, Q‐VD‐Oph and Z‐VAD‐FMK, but the level of NEDD4‐1 was still reduced in the presence of other apoptosis inhibitors, such as NQDI‐1 and Baxi (Fig. 2 *b*, Supporting Information Figs. S1*d* and S1*e*).

To evaluate the role of NEDD4‐1 in the sensitivity of MM cells to Bor, we altered NEDD4‐1 expression by transfecting HMCLs with NEDD4‐1 short hairpin RNAs (shRNAs) for KD experiments or HA‐NEDD4‐1 lentivirus vectors for OE experiments (Fig. 2 *c*, Supporting Information Figs. S1*f* and S1*g*). We designed three shRNA sequences and selected the b sequence for the following experiments (Supporting Information Fig. S1*h*). As shown in the cell growth curve, cell proliferation was increased in the NEDD4‐1‐KD cells compared to the shScramble controls, and cell proliferation was decreased in the NEDD4‐1‐OE cells compared to the EV‐transfected controls from Day 0 to Day 4 (Fig. 2 *d*). We next performed CCK‐8 assays and flow cytometry to determine the cell viability and apoptosis among the NEDD4‐1‐KD and NEDD4‐1‐OE MM cell lines treated with different concentrations of Bor for 24 hr. As shown in Figure 2 *e* and Supporting Information Figure S2*a*, NEDD4‐1‐KD HMCLs were less susceptible to Bor than the control group, and HA‐NEDD4‐1‐OE cells displayed more cell death in response to Bor treatment. Notably, the higher dose of Bor markedly widened the differences in the Bor response between the transfected cells and controls in RPMI8226 cells. HA‐NEDD4‐1 adenovirus was then transiently expressed with a different MOI, and cell viability in the presence of Bor gradually decreased as the level of HA‐NEDD4‐1 increased in MM cells (Fig. 2 *f*). However, the shRNA‐transfected RPMI8226 and NCI‐H929 cells exhibited no obvious increases in viability compared to the control when treated with another classic drug for the treatment of myeloma, melphalan (Supporting Information Fig. S2*b*). Additionally, we transfected the RPMI8226 and NCI‐H929 cells with the c sequence to rule out off‐target effects, and the NEDD4‐1 KD cells and those transfected with the b sequence were more resistant to Bor (Supporting Information Fig. S3*a*).

**Figure 3:**
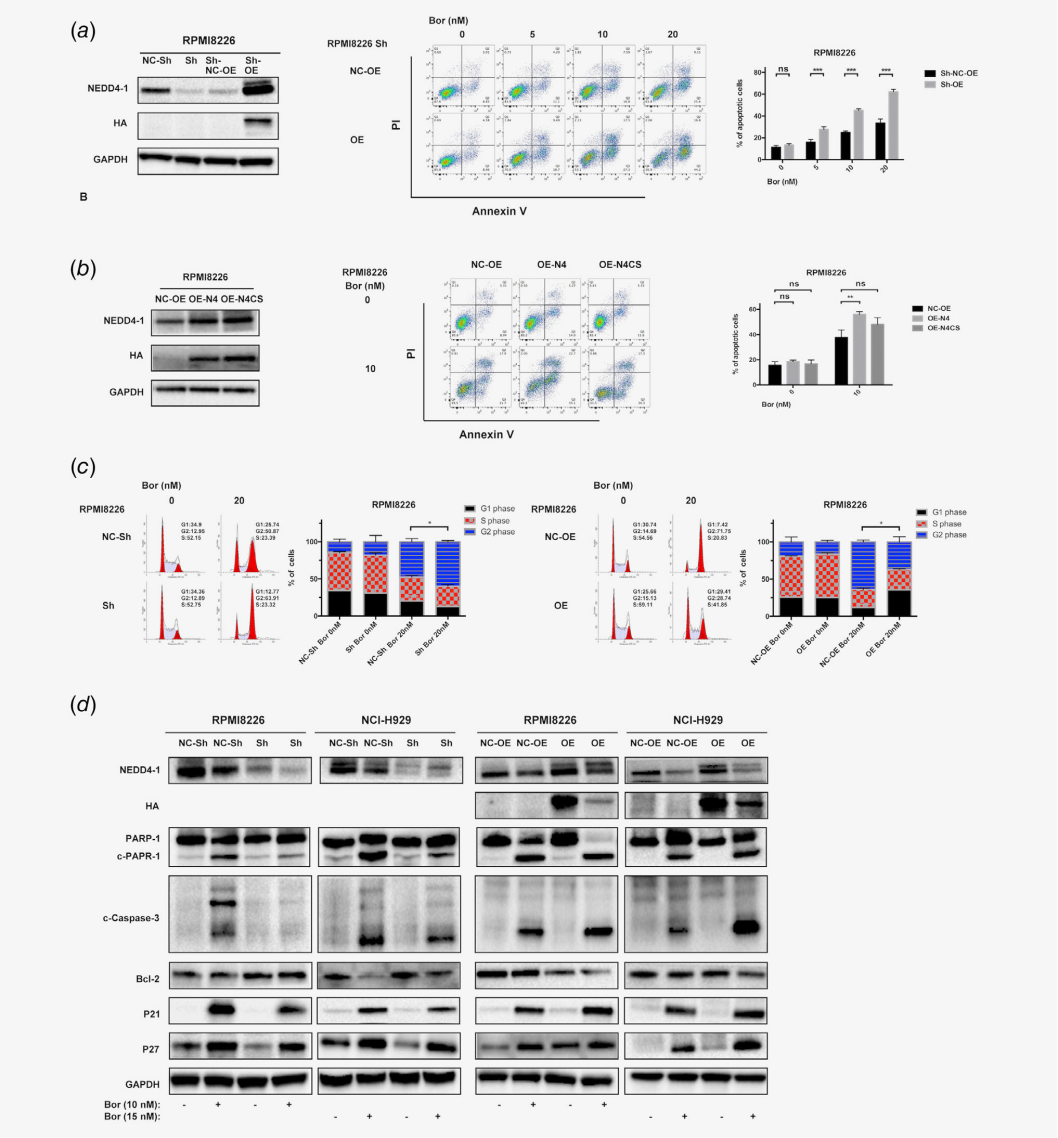
NEDD4‐1 mediates Bor sensitivity through apoptosis and the cell cycle and requires the HECT domain of NEDD4‐1. (*a*) Overexpression of HA‐NEDD4‐1 in NEDD4‐1‐deficient cells, and the cells were treated with Bor (0, 5, 10 and 20 nM) for 24 hr and detected by flow cytometry. The right histograms show the percentage of cells undergoing apoptosis. (*b*) HA‐NEDD4‐1 and HA‐NEDD4‐1‐CS were overexpressed in RPMI8226 cells, which were then treated with Bor (0 and 10 nmol/l) for 24 hr and detected by flow cytometry. The right histograms show the percentage of cells undergoing apoptosis. (*c*) The percentage of cells in the G1, S or G2 phase in NEDD4‐1 KD and OE RPMI8226 cell lines without or with 20 nM Bor treatment for 24 hr was detected by flow cytometry. Histograms show the percentage of HMCLs in the G_1_, S or G_2_ phase in three independent experiments. (*d*) NEDD4‐1 KD and OE HMCLs were treated with Bor for 24 hr. Cell lysates were collected, and the total levels of apoptosis and cell cycle‐related proteins were examined by Western blotting with the respective antibodies. Western Blot bands are derived from separate experiments but only one representative loading control is shown (*p < 0.05, **p < 0.01, ***p < 0.001). [Color figure can be viewed at http://wileyonlinelibrary.com]

To further verify the function of NEDD4‐1 in the Bor resistance of MM cells, an add‐back rescue experiment was performed. NEDD4‐1‐KD RPMI8226 cells were induced to overexpress lentivirus vector‐mediated HA‐NEDD4‐1. HA‐NEDD4‐1‐OE in the shRNA RPMI8226 cells induced more cell death than that observed in NEDD4‐1‐KD cells, and Bor treatment increased the value of the difference (Fig. 3 *a*). In addition, the Bor sensitivity caused by NEDD4‐1 appeared to be a direct effect of NEDD4‐1 E3 ligase activity because the enzyme‐dead HECT domain mutant, HA‐NEDD4‐1‐CS, failed to promote Bor sensitivity. Bor sensitivity was not obviously different between the EV‐transfected controls and the HA‐NEDD4‐1‐CS OE group, although the latter group showed slightly more cell death but without significance (Fig. 3 *b*, and Supporting Information Fig. S3*b*). Moreover, Figure 3 *c* and Supporting Information Figure S3*c* show an increased proportion of cells in the G_2_/M phase upon NEDD4‐1 KD, and the number of cells in the G_2_/M phase was decreased in the HA‐NEDD4‐1 OE group after treatment with Bor. The above results were also confirmed by further examining the changes in apoptosis and cell cycle‐related proteins through Western blotting (Fig. 3 *d*). Interestingly, there was a massive change in c‐Caspase‐3 but not much in P21, and we found that after changing the NEDD4‐1 gene, the difference in apoptosis was greater than the difference in cell cycles. The cell cycle only showed a difference at a certain drug concentration. We suspected that this may be the reason why p21 was no more obvious than caspase change.

### NEDD4‐1 binds with Akt and targets Akt for ubiquitination

**Figure 4:**
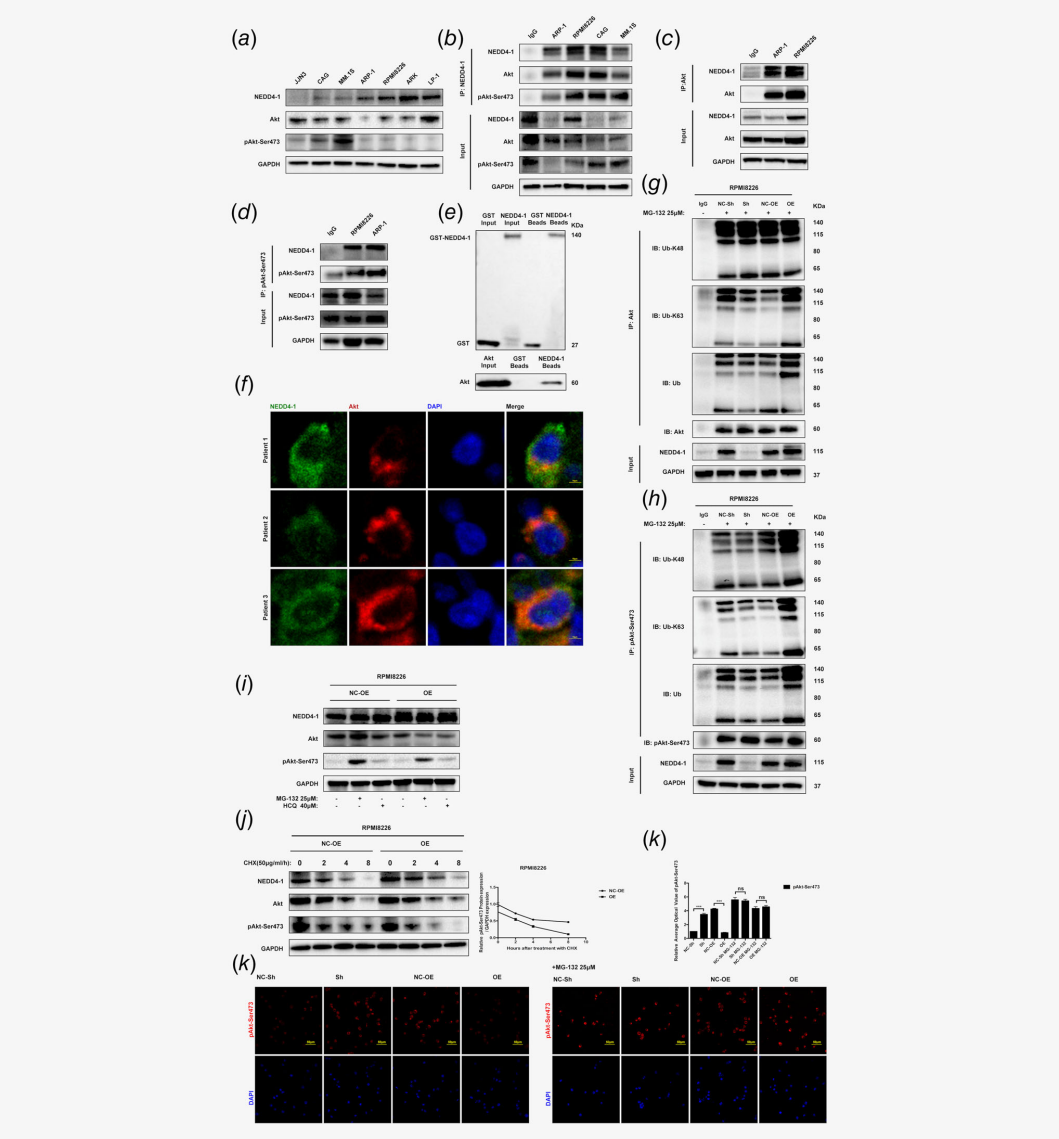
NEDD4‐1 regulates Akt ubiquitination. (*a*) The protein levels of NEDD4‐1, Akt and pAkt‐Ser473 in HMCLs. Whole‐cell extracts were analyzed by Western blotting with the indicated antibodies. (*b*) NEDD4‐1 immunoprecipitated endogenous Akt and pAkt‐Ser473. HMCL extracts were immunoprecipitated with antibodies against NEDD4‐1, along with the IgG control, followed by Western blotting with Akt and pAkt‐Ser473 antibodies. (*c*, *d*) Akt and pAkt‐Ser473 immunoprecipitates endogenous NEDD4‐1. ARP‐1 and RPMI8226 cells, along with the control, were lysed and immunoprecipitated with Akt and pAkt‐Ser473 antibodies, followed by incubation with the indicated antibodies. (*e*) The GST‐pull down assay showed the direct binding of NEDD4‐1 and Akt. The GST‐NEDD4‐1 fusion protein is approximately 140 kDa. The target protein can be detected in both the input and beads. The His‐Akt protein is approximately 60 kDa. His‐Akt can be detected in the supernatant of the bacterial lysates and in the supernatant of the GST‐NEDD4‐1 beads. GST alone was used as a negative control. GST‐NEDD4‐1 and His‐Akt were purified from *E. coli*. (*f*) Immunofluorescence staining (confocal microscopy) analysis of the subcellular colocalization of NEDD4‐1 and Akt in HMCLs. Nuclei were stained with DAPI. Scale bars, 10 μm. (*g*, *h*) NEDD4‐1 KD or OE RPMI8226 whole‐cell lysates (WCLs) were collected after 6 hr of MG‐132 (25 μm) treatment, and the protein levels of NEDD4‐1, Akt, pAkt‐Ser473 and GAPDH were evaluated by immunoblotting using their respective antibodies. Cell lysates were subjected to IP using anti‐Akt and anti‐pAkt‐Ser473 antibodies, and ubiquitinated Akt and pAkt‐Ser473 were detected with a related ubiquitin antibody. (*i*) NEDD4‐1 OE and EV‐transfected control RPMI8226 cells were treated with MG‐132 (25 μm) and hydroxychloroquine sulfate (HCQ; 40 μm) for 8 hr, and the protein levels of NEDD4‐1, Akt and pAkt‐Ser473 were evaluated by immunoblotting. (*j*) The pAkt‐Ser473 protein half‐life as impacted by NEDD4‐1 OE. RPMI8226 cells were treated with CHX at 50 μg/ml, and cell lysates were collected at the indicated times. pAkt‐Ser473 protein levels were assessed by immunoblotting. (*k*) Immunofluorescence staining analysis of the subcellular localization of pAkt‐Ser473 in the absence or presence of MG‐132 in ARP‐1 cells. The relative average optical (AO) value of pAkt‐Ser473 is shown in the upper right corner. Nuclei were stained with DAPI. Scale bars, 50 μm. Western Blot bands are derived from separate experiments but only one representative loading control is shown (*p < 0.05, **p < 0.01, ***p < 0.001). [Color figure can be viewed at http://wileyonlinelibrary.com]

To identify NEDD4‐1 E3 interaction partners and determine whether the Akt oncogene is significant in MM, the potential relationship between NEDD4‐1 and Akt in MM cells was explored. First, we examined the protein levels of NEDD4‐1 and Akt in HMCLs and found that the protein level of pAkt‐Ser473 was inversely correlated with the level of NEDD4‐1 (Fig. 4 *a*). We next evaluated the endogenous binding of two proteins by co‐IP and found that Akt and pAkt‐Ser473 were present in the NEDD4‐1 complex (Fig. 4 *b*) and HA (Supporting Information Fig. S4*a*) but not in the IgG control immunoprecipitation. Reciprocally, NEDD4‐1 was detected in the Akt and pAkt‐Ser473 immunoprecipitates (Figs. 4 *c* and 4 *d*). To test whether NEDD4‐1 directly interacted with Akt, we performed an *in vitro* GST pulldown assay using purified GST‐NEDD4‐1 and His‐tagged Akt. As shown in Figure 4 *e*, purified NEDD4‐1 was associated with purified Akt, indicating that NEDD4‐1 directly bound to Akt. Furthermore, we analyzed the subcellular localizations of these two proteins by immunofluorescence in primary MM cells. As shown in Figure 4 *f*, Akt and NEDD4‐1 were colocalized and distributed mainly in the cytosol.

To explore the role of NEDD4‐1 in Akt ubiquitination in MM, the lysates of NEDD4‐1‐KD and NEDD4‐1‐OE MM cells were immunoprecipitated with anti‐Akt and anti‐pAkt‐Ser473 antibodies, while Akt and pAkt‐Ser473 ubiquitination were detected with antibodies against ubiquitin. In RPMI8226 and ARP‐1 cells, NEDD4‐1 ubiquitinated Akt through K63 conjugation and not K48 ubiquitination but catalyzed both the K63‐linked and K48‐linked ubiquitination of pAkt‐Ser473, suggesting that NEDD4‐1 catalyzes the ubiquitination of pAkt‐Ser473, but not Akt, which may be involved in proteasomal degradation (Figs. 4 *g* and 4*h* and Supporting Information Figs. S4*b* and S4*c*).

To investigate the proteasomal *vs*. lysosomal degradation of pAkt‐Ser473, we performed experiments using MG‐132 and the lysosomal inhibitor HCQ. Consistent with the findings of previous studies,^31^ MG‐132 treatment led to the accumulation of pAkt‐Ser473, but chloroquine treatments did not. The pAkt‐Ser473 accumulation caused by MG‐132 treatment was attenuated in NEDD4‐1‐OE cells compared to EV‐transfected control cells. In contrast, no obvious effect of HCQ on pAkt‐Ser473 levels was observed in the control and NEDD4‐1‐OE cells (Fig. 4 *i* and Supporting Information Fig. S4*d*). To determine whether NEDD4‐1 affected the pAkt‐Ser473 protein half‐life, a CHX chase experiment was performed. As shown in Figure 4 *j* and Supporting Information Figure S4*e*, HA‐NEDD4‐1 OE caused a decreased basal level of endogenous pAkt‐Ser473 and a markedly shortened protein half‐life, as demonstrated by the treatment of cells for 0, 2, 4 and 8 hr with the protein biosynthesis inhibitor CHX. Furthermore, pAkt‐Ser473 was detected by immunofluorescence, and the expression level of NEDD4‐1 was decreased while pAkt‐Ser473 was increased in the cytosol, but this effect could be blocked by MG‐132 (Fig. 4 *k* and Supporting Information Fig. S4*f*).

### NEDD4‐1 potentiates Bor sensitivity in MM cells by degrading pAkt‐Ser473

**Figure 5:**
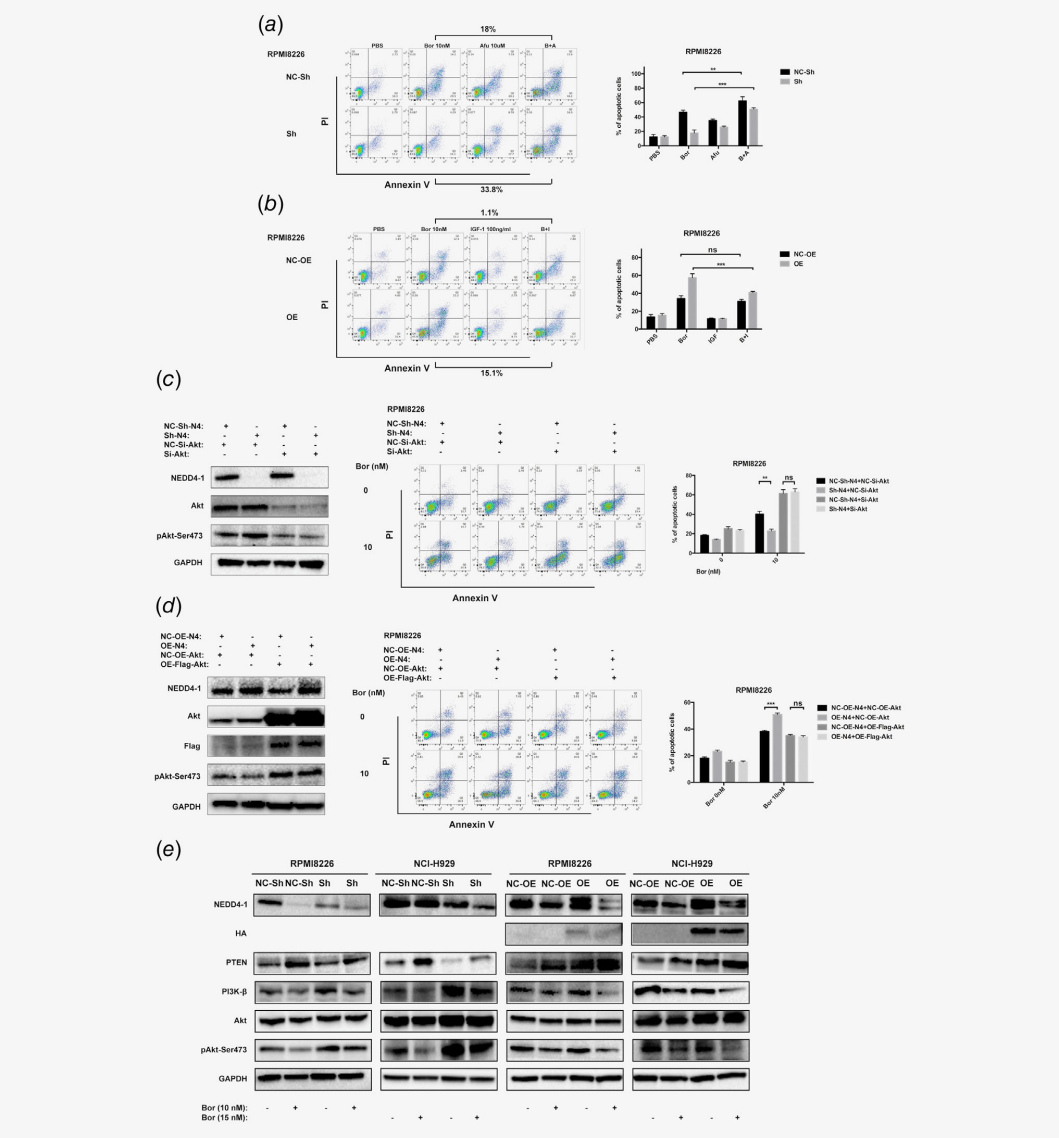
NEDD4‐1 modulates Bor resistance in MM cells by degrading pAkt‐Ser473. (*a*, *b*) NEDD4‐1 KD and OE RPMI8226 cells were seeded in 24‐well plates and incubated at 37°C for 24 hr with the indicated drugs. The cells were then analyzed by flow cytometry. Annexin V‐positive cells were considered apoptotic cells. The right histograms show the percentage of cells undergoing apoptosis. (*c*) NEDD4‐1 KD and Akt siRNA in RPMI8226 cells were treated with Bor (0 and 10 nmol/l) for 24 hr and detected by flow cytometry. The right histograms show the percentage of cells undergoing apoptosis. (*d*) NEDD4‐1 OE and Flag‐Akt in RPMI8226 cells were treated with Bor (0 and 10 nmol/l) for 24 hr and detected by flow cytometry. The right histograms show the percentage of cells undergoing apoptosis. (*e*) NEDD4‐1 KD and OE RPMI8226 and NCI‐H929 cells were treated with or without Bor for 24 hr. Cell lysates were collected, and the total levels of NEDD4‐1, HA, PTEN, PI3K‐β, Akt, pAkt‐Ser473 and GAPDH were examined by Western blotting with their respective antibodies. Western Blot bands are derived from separate experiments but only one representative loading control is shown (*p < 0.05, **p < 0.01, ***p < 0.001). [Color figure can be viewed at http://wileyonlinelibrary.com]

The elevated K48‐linked ubiquitination and degradation of pAkt‐Ser473 that we observed upon NEDD4‐1 OE prompted us to investigate whether the change in drug sensitivity induced by NEDD4‐1 could be blocked by the Akt inhibitor Afu or the Akt upstream mediator insulin‐like growth factor‐1 (IGF‐I).^23^, ^32^ First, we evaluated the effects of Afu and IGF‐I on pAkt‐Ser473 levels in MM cells (Supporting Information Fig. S5*a*). In a flow cytometric analysis of RPMI8226 cells, the apoptotic cells transfected with shScramble were increased after Bor treatment for 24 hr compared to NEDD4‐1 KD cells. Afu treatment reduced the live‐cell fractions of both the NEDD4‐1‐KD and shScramble control cells, but the effect was more prominent in the NEDD4‐1 KD cells than in the shScramble control cells (33.8% *vs*. 18% reduction in live cells; Fig. 5 *a*). Consistently, NEDD4‐1 OE in MM cells resulted in greater Bor sensitivity compared to that of the control cells. IGF‐I promoted cell growth. Bor‐treated HA‐NEDD4‐1‐OE cells were more responsive to IGF‐I than the control group (15.1% *vs*. 1.1% increase in live cells; Fig. 5 *b*). Furthermore, we transfected NEDD4‐1‐KD RPMI8226 cells with Akt siRNA, and the Bor resistance caused by NEDD4‐1‐KD essentially disappeared (Fig. 5 *c*). After transfection with Flag‐Akt lentivirus in NEDD4‐1‐OE RPMI8226 cells, the effect of greater sensitivity to Bor induced by NEDD4‐1 was abolished by the simultaneously increased level of pAkt‐Ser473 (Fig. 5 *d*). These results suggested that pAkt‐Ser473 may be a novel NEDD4‐1‐interacting protein in MM and that its expression is required for NEDD4‐1‐mediated Bor resistance.

Akt is the hub of PTEN/PI3K/Akt signaling. To determine whether altered NEDD4‐1 expression changes Akt signaling, we investigated the steady‐state levels of PTEN, PI3K and Akt in MM cells harboring the control, NEDD4‐1 shRNA or HA‐NEDD4‐1 lentivirus vector. Our results indicated that the activation of PI3K and pAkt‐Ser473 expression was promoted but the expression of PTEN was markedly diminished in cells transfected with shRNA compared to the control group cells upon stimulation with Bor. Consistently, the HA‐NEDD4‐1‐transfected cells exhibited an inactivation of the PTEN/PI3K/Akt signaling pathway (Fig. 5 *e*).

### Therapeutic evaluation of NEDD4‐1 in the MM xenograft mouse model

**Figure 6:**
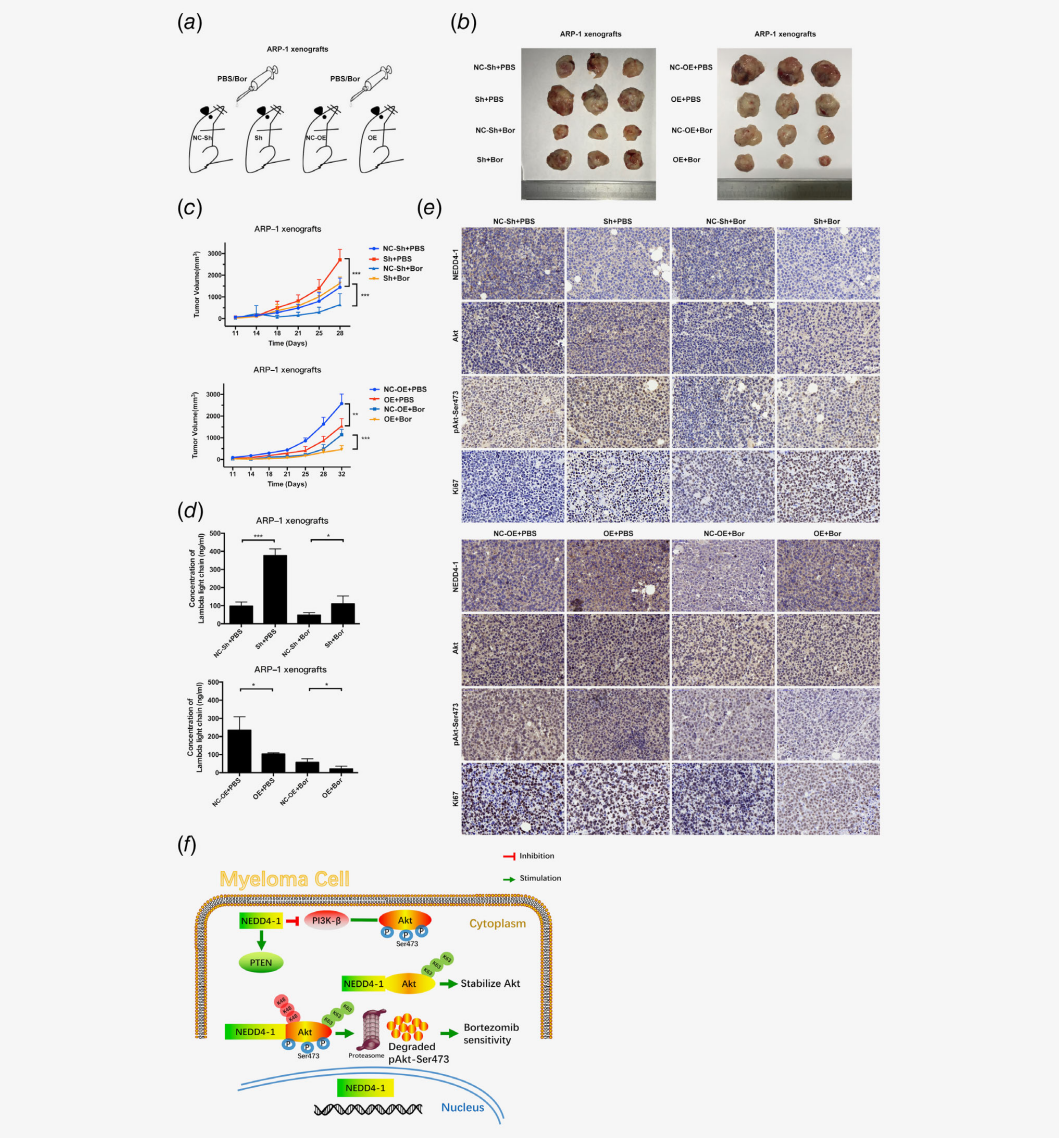
Therapeutic evaluation of NEDD4‐1 *in vivo*. (*a*) Schematic showing the experimental design of the tumor model. (*b*) Xenografts were excised and imaged at Day 28. (*c*) A total of 5 × 10^6^ NEDD4‐1 KD or OE ARP‐1 cells were subcutaneously injected into NOD‐SCID mice (*n* = 24). The sizes of the tumors were measured, and PBS or Bor (0.5 mg/kg) was injected every 3–4 days when the established tumors reached approximately 100–130 mm^3^ on Day 11. The results shown are the mean tumor volume ± SEM. Student's *t*‐test was applied for pairwise statistical comparisons. (*d*) The peripheral blood supernatants of ARP‐1 xenograft NOD/SCID mice were collected to detect lambda light chain levels using ELISA. (*e*) Immunohistochemistry analyses with NEDD4‐1, Akt, pAkt‐Ser473 and Ki67 antibodies. Magnification, 200×. Scale bars, 50 μm. (*f*) Schematic for the regulatory relationship between NEDD4‐1 and Akt signaling in MM cells (*p < 0.05, **p < 0.01, ***p < 0.001). [Color figure can be viewed at http://wileyonlinelibrary.com]

Having shown the potential antimyeloma effect of NEDD4‐1 *in vitro*, we next examined the effects of NEDD4‐1 *in vivo* using an ARP‐1 xenograft NOD/SCID mouse model. Bor or PBS was administered by intraperitoneal injection every 3–4 days when the tumors reached approximately 100–130 mm^3^ (Fig. 6 *a*). The tumor growth patterns of the mice showed that Bor treatment inhibited tumor growth from days 11 to 32, and the mice were sacrificed when the tumor reached approximately 3,000 mm^3^. The tumor size was significantly increased in the NEDD4‐1 KD group (2,704 mm^3^) compared to the control group (1,441 mm^3^) on Day 28. Notably, on Day 28, the tumor volumes were relatively larger (1,628 mm^3^) in the KD group than in the control group (644 mm^3^) with Bor treatment (Figs. 6 *b* and 6*c*). Since Bor treatment increased the difference between the KD group and the control group, the ratio of the KD *versus* control group increased from 1.87 to 2.52. We examined the tumor burden by measuring the lambda light chain levels in the peripheral blood supernatants of ARP‐1 xenograft NOD/SCID mice. The median level of soluble lambda light chains was higher in the NEDD4‐1 KD group than in the control group, as measured by ELISA (Fig. 6 *d*). As expected, the tumor burden measured in HA‐NEDD4‐1 OE ARP‐1 xenograft NOD/SCID mice was significantly decreased compared to that in the control (Figs. 6 *b–*6*d*). During the treatment period, no significant change in body weight was observed. Additionally, immunohistochemical analysis showed that NEDD4‐1‐repressed tumors exhibited more negative staining for c‐PARP‐1, cleaved Caspase‐3, P21, PTEN and TUNEL, yet more positive staining for pAkt‐Ser473 and Ki67, while the HA‐NEDD4‐1 OE group showed the opposite results (Fig. 6 *e* and Supporting Information Fig. S6*a*). These results demonstrated the efficacy of NEDD4‐1 repression in inducing tumor growth and attenuating the Bor effect.

## Discussion

Aberrant NEDD4‐1 expression has been observed in various tumors. While NEDD4‐1 is an oncogene in most cancers,^16^, ^17^, ^18^, ^19^, ^20^, ^21^, ^22^, ^33^, ^34^ it can also act as a tumor suppressor in some tumors^14^, ^15^, ^35^, ^36^; however, the role of NEDD4‐1 in MM remains unclear. In our study, low NEDD4‐1 expression was closely related to worse outcomes among MM patients. HA‐NEDD4‐1 OE, but not the enzyme‐dead NEDD4‐1‐C867S mutant, sensitized MM cells to Bor, suggesting that an NEDD4‐1 enzymatic activator may be proposed as a novel targeted therapy to overcome the Bor resistance mediated by low NEDD4‐1 activity.

NEDD4 family members commonly affect tumor growth, including that of MM. WWP2, a member of the NEDD4 family, increases in the presence of gamabufotalin and plays an important role in inhibiting the growth of MM cells and apoptosis in vitro.^37^ MM patients with methylated Smurf2 promoters are correlated with an increased risk of death, advanced stages and a reduced risk of extramedullary disease.^38^ The increased availability of effective therapies has been accompanied by an increase in acquired drug resistance. The main forms of drug resistance include the blockade of apoptosis caused by numerous anticancer drugs, the activation of detoxifying drug and DNA‐damage repair mechanisms, changes in the cell cycle and changes in checkpoints.^39^ Here, we showed that NEDD4‐1 deficiency led to less apoptosis and G2/M phase cell arrest, which provides proof of Bor resistance in NEDD4‐1 KD HMCLs. Consistently, the expression levels of the apoptosis‐related proteins c‐Caspase‐3 and PARP‐1 and the cell cycle proteins P21 and P27 were downregulated, and HA‐NEDD4‐1 OE correlated with the opposite results (Fig. 3).

We found that NEDD4‐1 KD induced less sensitivity to Bor among MM cells (Fig. 2 *e*), but MM cells underwent apoptosis accompanied by a decrease in NEDD4‐1 when treated with Bor (Fig. 2 *a* and Supporting Information Fig. S1*b*), which seems contradictory. Apoptosis induced by various stimuli, such as radiation and drug treatment, has been suggested to cause NEDD4‐1 to be cleaved, and this cleavage was inhibited by an inhibitor of caspase‐3‐like proteases. The NEDD4‐1 cleavage products do not induce apoptosis.^40^ Therefore, we added pan‐caspase inhibitors, Q‐VD‐Oph or Z‐VAD‐FMK, to the Bor‐treated MM cells. Indeed, Bor reduced the level of NEDD4‐1, but this phenomenon disappeared after the addition of the pan‐caspase inhibitors (Fig. 2 *b* and Supporting Information Figs. S2*d* and S2*e*). In addition, other noncaspase inhibitors of apoptosis did not restore the expression of NEDD4‐1, and Bor treatment did not change the localization of NEDD4‐1 (Supporting Information Figs. S2*c*–S2*e*). Thus, the gradual decrease in NEDD4‐1 with Bor treatment may be due to the apoptosis of the caspase pathway induced by Bor to some extent. Moreover, we reasoned that Bor caused a decrease in the expression of NEDD4‐1, which may be the cause of Bor resistance, in other words, NEDD4‐1 may be the target of Bor to exert efficacy. When NEDD4‐1 expression was high, Bor exerted its efficacy, and NEDD4‐1 gradually decomposed. Under the continuous action of Bor, MM cells gradually became insensitive to Bor, while NEDD4‐1 levels gradually decreased. After NEDD4‐1 OE, MM cells were resensitized to Bor.

Akt has been reported to play a crucial role in the occurrence and development of MM. There is currently an intense effort towards developing inhibitors targeting Akt for the treatment of MM.^23^ Frequently, multiple E3 ligases regulate Akt substrates, such as TRAF3,^41^ TRAF6^27^ and Skp2.^42^ Since TRAF6 has been identified as a major Akt ubiquitin ligase with implication also in MM and as a potential therapeutic target,^43^ we also found that TRAF6 is positively correlated with Bor resistance (Fig. 1 *l*). Fan showed that NEDD4‐1 can ubiquitinate pAkt‐Ser473 in the IGF‐I response.^28^ However, no direct evidence has demonstrated a role for the NEDD4‐1‐Akt axis in MM. We found that NEDD4‐1 directly binds with Akt, and NEDD4‐1 could ubiquitinate Akt and degrade pAkt‐Ser473 *via* the proteasome in MM, which may be due to the K63 linkages required for Akt ubiquitination, whereas K48 linkages target pAkt‐Ser473 for proteasomal degradation.^10^ Since Bor and MG‐132 are both proteasome inhibitors, they are expected to lead to similar results if NEDD4‐1 promotes pAkt‐Ser473 degradation, which should be rescued by both chemicals. However, the opposite results were observed: Bor reduced pAkt‐Ser473 (Fig. 5 *e*), while MG‐132 increased pAkt‐Ser473 (Fig. 4 *i*). The key difference between Bor and MG‐132 was that Bor caused a significant decrease in NEDD4‐1 at both the mRNA and protein levels (Fig. 2 *a*), while MG‐132 did not cause changes in NEDD4‐1 levels (Fig. 4 *i*). Therefore, we hypothesize that some key molecules activated by the Bor‐induced downregulation of NEDD4‐1, but not MG‐132, may reflect an alternative mechanism for Bor responsiveness *via* pAkt downregulation.

Elevated pAkt‐Ser473 upon NEDD4‐1 KD rendered cells resistant to Bor. Cells overexpressing HA‐NEDD4‐1 lost their ability to increase pAkt‐Ser473, and these MM cells became sensitive to Bor. Notably, NEDD4‐1‐KD cells were more sensitive to Bor with Akt‐inactivating treatments than shScramble control cells, and HA‐NEDD4‐1‐OE cells were more responsive to Bor with Akt‐activating treatments, suggesting that the changes in NEDD4‐1‐induced drug sensitivity were a result of Akt alterations (Fig. 5). In addition, the increase in pAkt‐Ser473 with Afu treatment was consistent with the findings of a previous study reporting that ATP‐competitive Akt kinase inhibitors show a concentration (dose and time)‐dependent feedback increase in Akt phosphorylation.^44^, ^45^ This study also showed that the feedback increase was more obvious in the shRNA cells than in the shScramble controls (Supporting Information Fig. S5*a*). These observations are consistent with those of previous studies reporting that high levels of pAkt‐Ser473 activation may promote cell proliferation and that low levels of pAkt‐Ser473 activation cause cell growth inhibition.^46^ Furthermore, the intracellular Akt signaling pathway is reportedly significant in modulating a vast array of cellular processes involved in the cell cycle and apoptosis.^47^, ^48^ The actions of NEDD4‐1, as a tumor suppressor gene, may have consequences in MM due to the ubiquitination of Akt and a decrease in Akt phosphorylation. The regulation of pAkt‐Ser473 by NEDD4‐1 has significant implications in our understanding of Akt in MM. Our working model for the NEDD4‐1/Akt interaction and its underlining biology is shown in Figure 6 *f*. Further studies are needed to identify the interaction site of NEDD4‐1 and Akt and characterize their relationship in a large number of primary MM samples.

NEDD4‐1 was the first identified PTEN E3 ligase. Wang *et al*. found that NEDD4‐1 is a negative regulator of PTEN and promotes PTEN to be poly‐ubiquitinated in the cell and degraded. Increasing levels of NEDD4‐1 significantly reduced PTEN expression and potentiated cell proliferation and prostate/bladder tumor formation, suggesting an oncogenic role of NEDD4‐1 in regulating PTEN functions.^49^, ^50^ Besides degradation, NEDD4‐1 can also monoubiquitinate PTEN to regulate it into the nucleus.^35^However, subsequent studies have shown no difference in the stability and localization of PTEN in two different strains of NEDD4‐1‐deficient mice.^51^ In our study, we found that Bor increased the expression of PTEN in the shRNA group but to a lesser extent than in the controls. In contrast, Huang *et al*. found that the depletion of NEDD4‐1 reduces pAKT levels, increases PTEN levels and suppresses the growth and migration abilities of HCC cells.^52^ The protein level of NEDD4‐1 in Hep3B is higher than that of many MM cells. NEDD4‐1 plays different roles in the above results, possibly due to differences in research systems or cell contexts, similar to Plk, which has dual roles in cancers.^53^

Intense efforts are currently underway to address acquired resistance to Bor, which results in recurrence and poor prognoses for patients with MM during long‐term Bor treatment.^3^, ^54^, ^55^ For the first time, we identified a novel gene, NEDD4‐1, that may play a key role in the development of Bor resistance in MM. Specifically, NEDD4‐1 KD in HMCLs showed no obvious antiapoptotic effects compared to the controls when treated with another drug, namely, melphalan. We tentatively propose that NEDD4‐1 can serve as a response marker for Bor‐resistance therapy in MM in addition to other drug‐resistance therapies. In other words, a decreased level of NEDD4‐1 in MM patients may indicate a poor response to Bor and induce Bor resistance. Further investigations of the role of NEDD4‐1 in MM should include evaluations of new target substrates and the translation of the NEDD4‐1 enzymatic activator into clinical treatment.

## Supporting information


**Appendix S1**: Supporting Information

## Data Availability

The data that support the findings of our study are available from the corresponding author upon reasonable request. Further details are provided in the supplementary methods and reagents.
