# Supplementary material for: The NEDD4‐1 E3 ubiquitin ligase: A potential molecular target for bortezomib sensitivity in multiple myeloma
Source: Int J Cancer. 2019 Aug 24;146(7):1963–78. doi: 10.1002/ijc.32615 (PMC7027789; doi:10.1002/ijc.32615)

**Supplementary Information**

**Methods and reagents**

**Cell culture and transfection**

HMCLs were cultured in RPMI-1640 medium containing L-glutamine (Corning Cellgro, USA) and supplemented with 10% fetal bovine serum (Thermo Fisher Scientific, Gibco, USA) at 37°C in a humidified atmosphere with 5% CO_2_. For stable NEDD4-1 KD and HA-NEDD4-1 expression, cells were selected in culture media containing puromycin (2 μg/ml) for 2 weeks. For stable Akt expression, HMCLs were transfected with Flag-Akt lentiviral particles and selected with G418 (100 µg/ml) for 2 weeks (ABM Inc., Vancouver, Canada).

**Cell proliferation, cell apoptosis and cell cycle assays**

CCK-8 assay was used to measure MM cell proliferation and viability. MM cells were seeded in 96-well plates and treated with the indicated concentrations of drugs. After incubation for the indicated times, the cells were treated with CCK-8 solution and incubated for another 2 h. Then, absorbance was measured at 450 nm using a microplate reader. To assess the impacts of Bor or Mel on apoptosis and the cell cycle of MM cells, MM cells were seeded in 24/12-well plates for 24 h. Then, flow cytometry was used to assess the cell cycle distribution and apoptosis, and the results were analysed with FlowJo 7.6.1 software.

**Western blotting analysis and co-IP**

Western blotting was performed as previously reported. Briefly, MM cell lines were harvested and washed twice with PBS, and total protein was extracted with lysis buffer containing a mixture of protease and phosphatase inhibitors. The supernatants containing total cellular protein were collected for Western blotting. The immunoblots were incubated with specific primary antibodies overnight at 4°C. The next day, the membranes were washed with TBS-T and incubated with HRP-conjugated anti-rabbit or anti-mouse antibodies at room temperature for 1 h. The signals were detected using the ChemiDoc MP Imaging System with an enhanced chemiluminescence detection kit. To immunoprecipitate exogenously expressed HA-tagged proteins, the precleared cell lysates were incubated with HA antibody for 3 h followed by incubation with beads at 4°C overnight with rotation. The beads were washed three times with lysis buffer, and the immunoprecipitation complexes were subjected to SDS-PAGE.

**Supplementary Figures S1-S9 figures and legends:**

**Supplementary Figure S1. Alteration in NEDD4-1 expression in MM cells.** (a) Immunofluorescence staining (phase contrast microscopy and confocal microscopy) analysis of the negative control staining of NEDD4-1and CD138 in MM patients. Nuclei were stained with DAPI. Scale bars, 50 μm. (b) ARP-1 cells were treated with different concentrations of Bor (0, 5, 10, and 20 nM) for 24 h or 2 nM Bor for different durations (0, 6, 12, and 24 h). The mRNA and protein levels of NEDD4-1 were evaluated by RT-PCR and Western blotting. (c) Western blotting analysis of the subcellular localization of NEDD4-1 in RPMI8226 cells with or without Bor (10 nM). Cyto refers to the cytoplasm, and Nuc refers to the nucleus. (d) RPMI8226 cells were treated with or without Bor (10 nM) or NQDI-1 (15 μm), Baxi (15 μm), or Z-VAD-FMK (40 mM). Whole-cell extracts were analysed by Western blotting with NEDD4-1 and GAPDH antibodies. NQDI-1: ASK1 (apoptosis signal-regulated kinase 1) inhibitor, Baxi: A Bax-mediated apoptosis inhibitor. (e) MM.1S cells were treated with or without Bor (10 nM) or Q-VD-Oph (50 mM). Whole-cell extracts were analysed by Western blotting with NEDD4-1, PARP-1, c-Caspase, and GAPDH antibodies. (f) Verification of the effect of NEDD4-1 KD or OE in HMCLs. RT-PCR showed an 80% average change in the NEDD4-1 level. (g) The efficiency of NEDD4-1 KD and OE transfection was detected by fluorescence microscopy. The lentiviral particles containing green fluorescent protein directed against human NEDD4-1 (KD and OE) and the shScramble control. (h) Three independent shNEDD4-1 KD procedures in RPMI8226 cell lines resulted in a significant decrease in NEDD4-1 levels as determined by immunoblots. sh-b was then used in the following experiments. Western Blot bands are derived from separate experiments but only one representative loading control is shown. */#/&P < 0.05, **/##/&&P < 0.01, ***/###/&&&P < 0.001.

**Supplementary Figure S2. NEDD4-1 KD mediates Bor but not melphalan resistance in MM cells.** (a) NEDD4-1 KD or OE HMCLs were treated with the indicated concentration of Bor. After 24 h of incubation at 37°C, cell viability was measured by CCK-8 assay, and cell apoptosis was detected by flow cytometry. Annexin V-positive cells were considered apoptotic cells. The right histograms show the percentage of cells undergoing apoptosis. “ns” refers to “non-significance”. (b) NEDD4-1 KD HMCLs were seeded in 96-well plates with the indicated concentration of Mel, and after 24 h of incubation at 37°C, cell viability was measured by CCK-8 assay. The middle and right panels show the apoptotic cells using flow cytometry. *P < 0.05, **P < 0.01, ***P < 0.001.

**Supplementary Figure S3. NEDD4-1, but not the enzyme-dead NEDD4-1-C867S mutant, induces Bor sensitivity, and NEDD4-1 contributes to less G2/M phase cell cycle arrest.** (a) NEDD4-1 KD (c sequence) HMCLs were seeded in 96-well plates with the indicated concentration of Bor, and after 24 h of incubation at 37°C, cell viability was measured by CCK-8 assay. (b) HA-NEDD4-1 OE and HA-NEDD4-1-CS OE in CAG cells were treated with Bor (0 and 5 nmol/L) for 24 h and detected by flow cytometry. The right histograms show the percentage of cells undergoing apoptosis. (c) The percentage of cells in the G_1_, S, or G_2_ phase in NEDD4-1 KD and OE NCI-H929 cells without or with 7.5 nM Bor treatment for 24 h were detected by flow cytometry. Histograms show the percentage of HMCLs in the G_1_, S, or G_2_ phases in three independent experiments. Western Blot bands are derived from separate experiments but only one representative loading control is shown. *P < 0.05, **P < 0.01, ***P < 0.001.

**Supplementary Figure S4. NEDD4-1 interacts with Akt.** (a) HA immunoprecipitated endogenous Akt. ARP-1 cells were transfected with HA-NEDD4-1, along with a control. Cells were lysed and immunoprecipitated with HA antibody, followed by incubation with the indicated antibodies. (b, c) NEDD4-1 KD or OE ARP-1 whole-cell lysates (WCLs) were collected following 6 h of MG-132 (25 μm) treatment, and the protein levels of NEDD4-1, Akt, pAkt-Ser473 and GAPDH were evaluated by immunoblotting using their respective antibodies. Cell lysates were subjected to immunoprecipitation using anti-Akt and anti-pAkt-Ser473 antibodies, and ubiquitinated Akt and pAkt-Ser473 were detected with a related ubiquitin antibody. (d) NEDD4-1 OE and EV-transfected control ARP-1 cells were treated with MG-132 (25 μm) and HCQ (40 μm) for 8 h, and the protein levels of NEDD4-1, Akt and pAkt-Ser473 were evaluated by immunoblotting. (e) The pAkt-Ser473 protein half-life was impacted by NEDD4-1 OE. ARP-1 cells were treated with CHX at 50 μg/ml, and cell lysates were collected at the indicated times. pAkt-Ser473 protein levels were assessed by immunoblotting. (f) Immunofluorescence staining analysis of the negative control staining of pAkt-Ser473 in ARP-1 cells. Nuclei were stained with DAPI. Scale bars, 50 μm. Western Blot bands are derived from separate experiments but only one representative loading control is shown.

**Supplementary Figures S5. The effects of Afu or IGF-1 on the pAkt-Ser473 levels of NEDD4-1 KD or OE RPMI8226 cells.** (a) Western blotting analysis showed the effects of Afu or IGF-1 on the pAkt-Ser473 levels of NEDD4-1 KD or OE RPMI8226 cells, respectively. Whole-cell extracts were subjected to Western blotting with the indicated antibodies. Western Blot bands are derived from separate experiments but only one representative loading control is shown.

**Supplementary Figure S6. Immunohistochemical analysis in xenograft models of MM.** (a) Immunohistochemistry analyses with anti c-PARP-1, cleaved Caspase-3, P21, PTEN, TUNEL, and HE antibodies. Magnification, ×200. Scale bars, 50 μm. *P < 0.05, **P < 0.01, ***P < 0.001.

**Supplementary Figure S7-9.** Protein bands throughout the manuscript were analyzed with Image Lab software. *P < 0.05, **P < 0.01, ***P < 0.001.


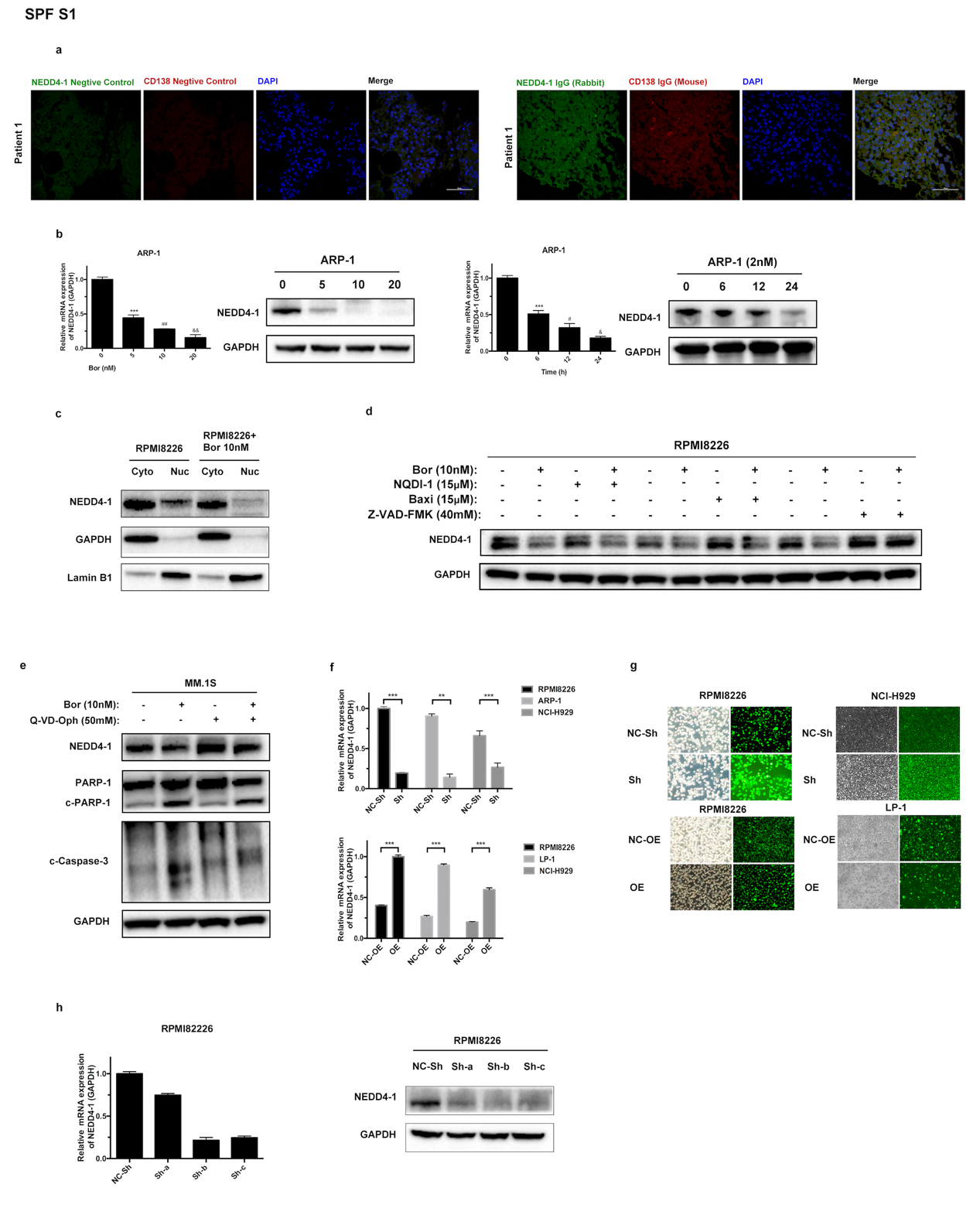


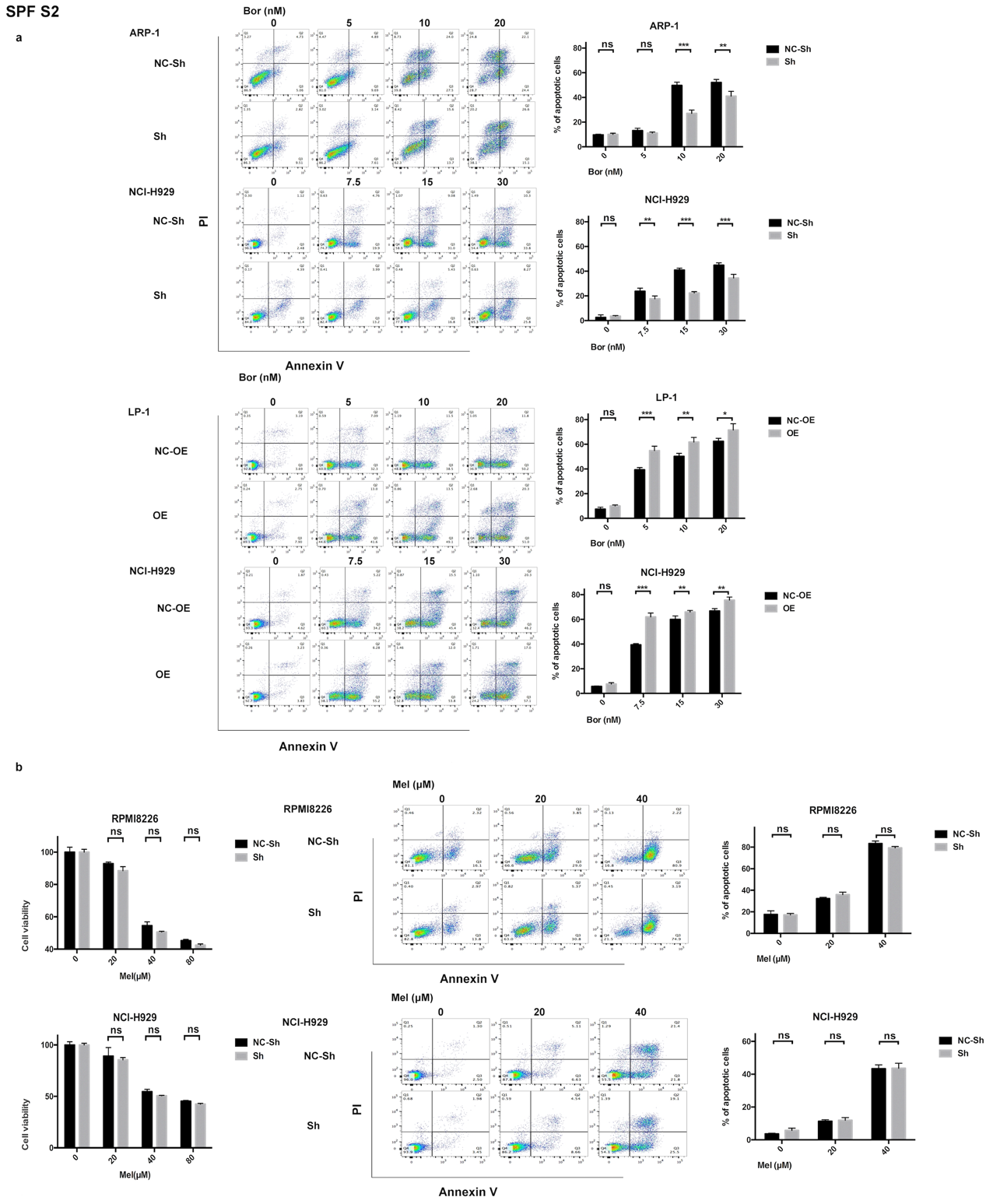


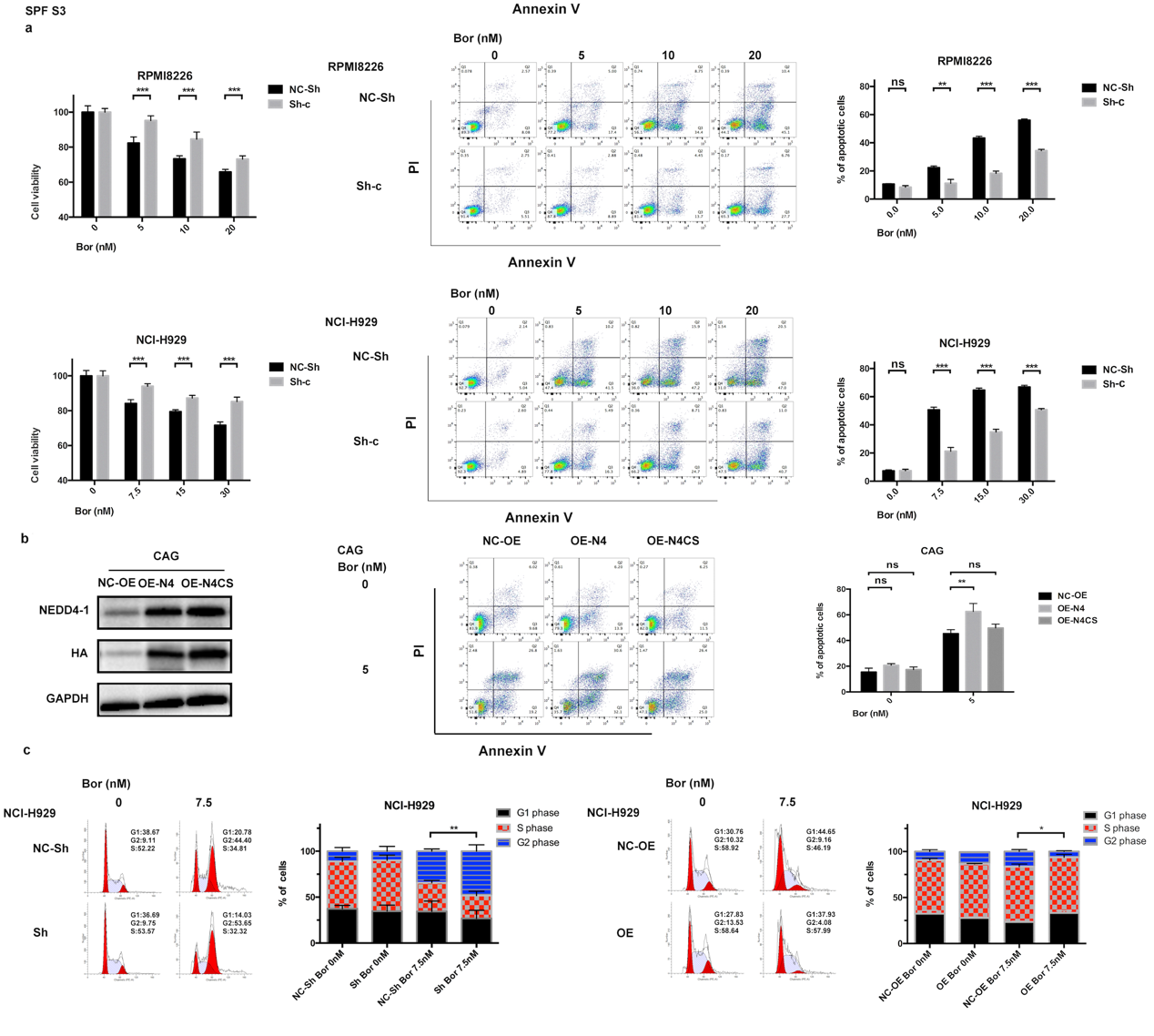


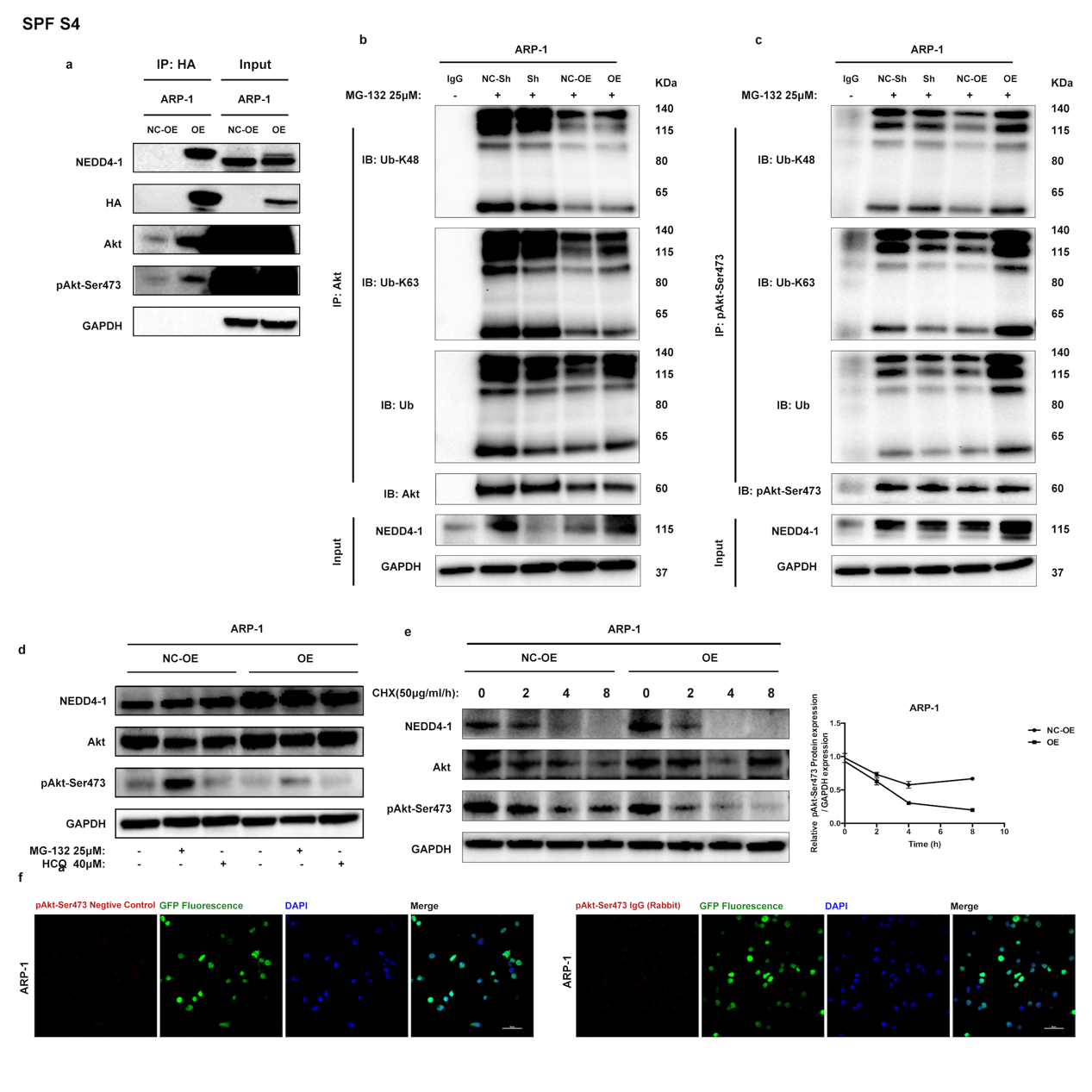


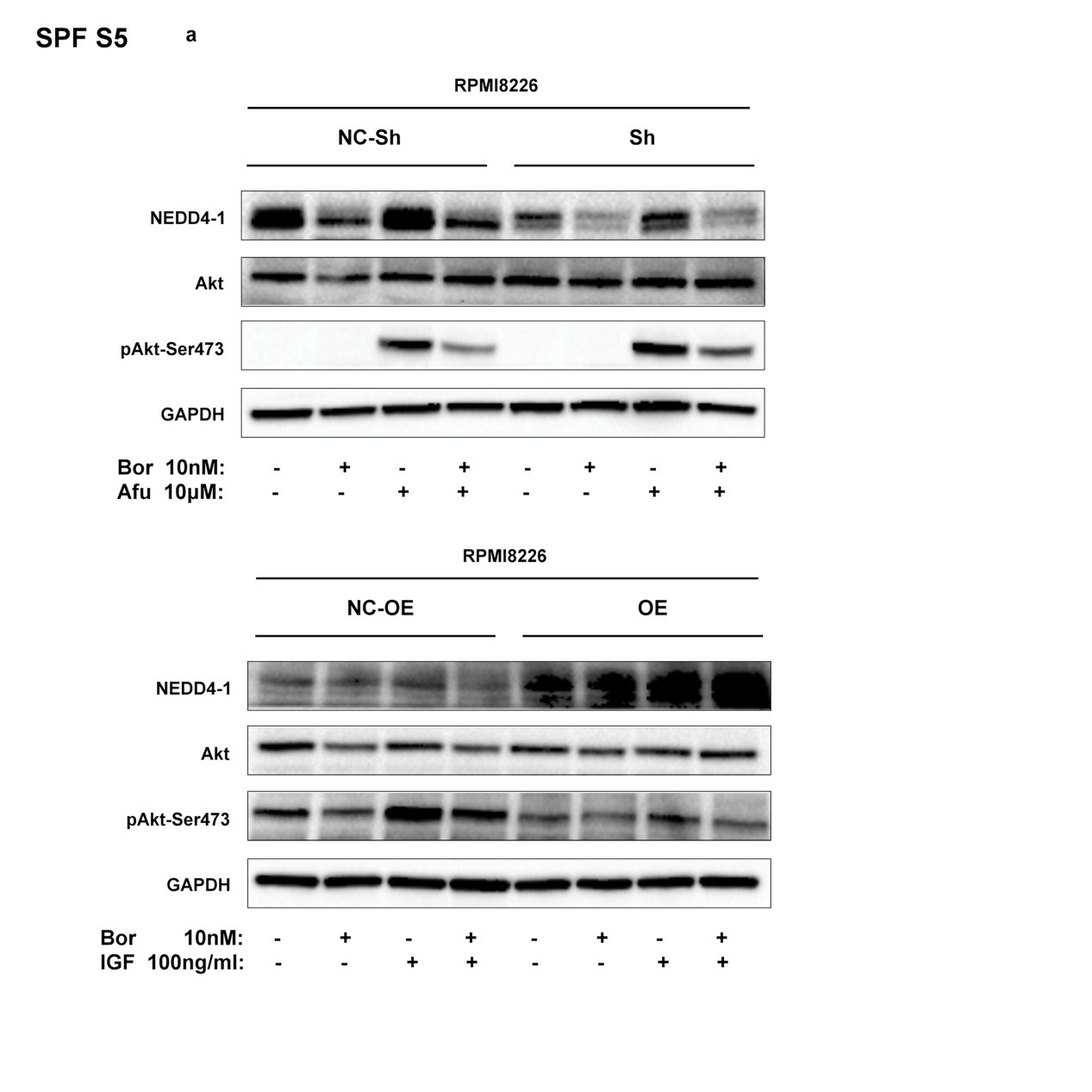


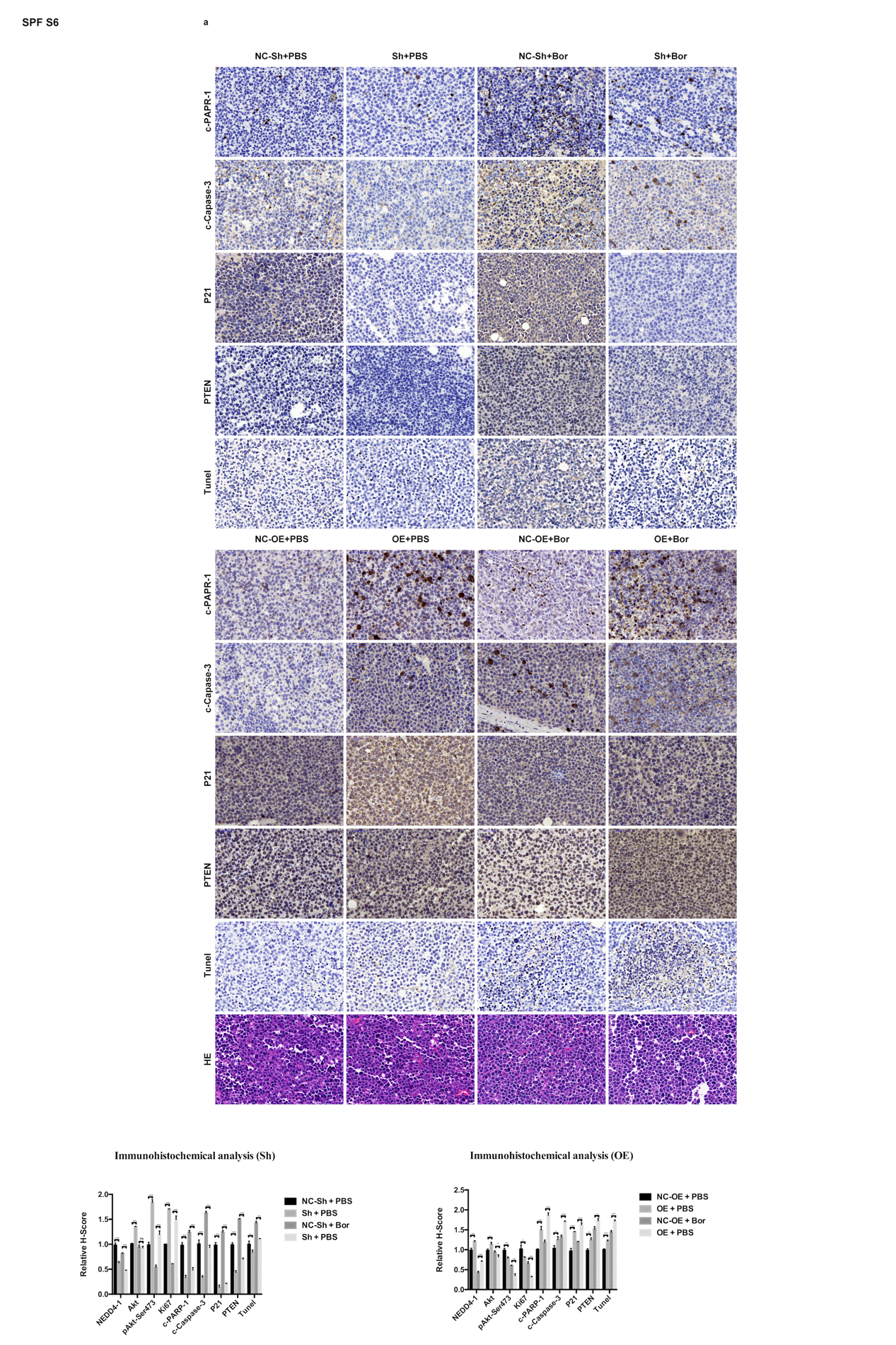


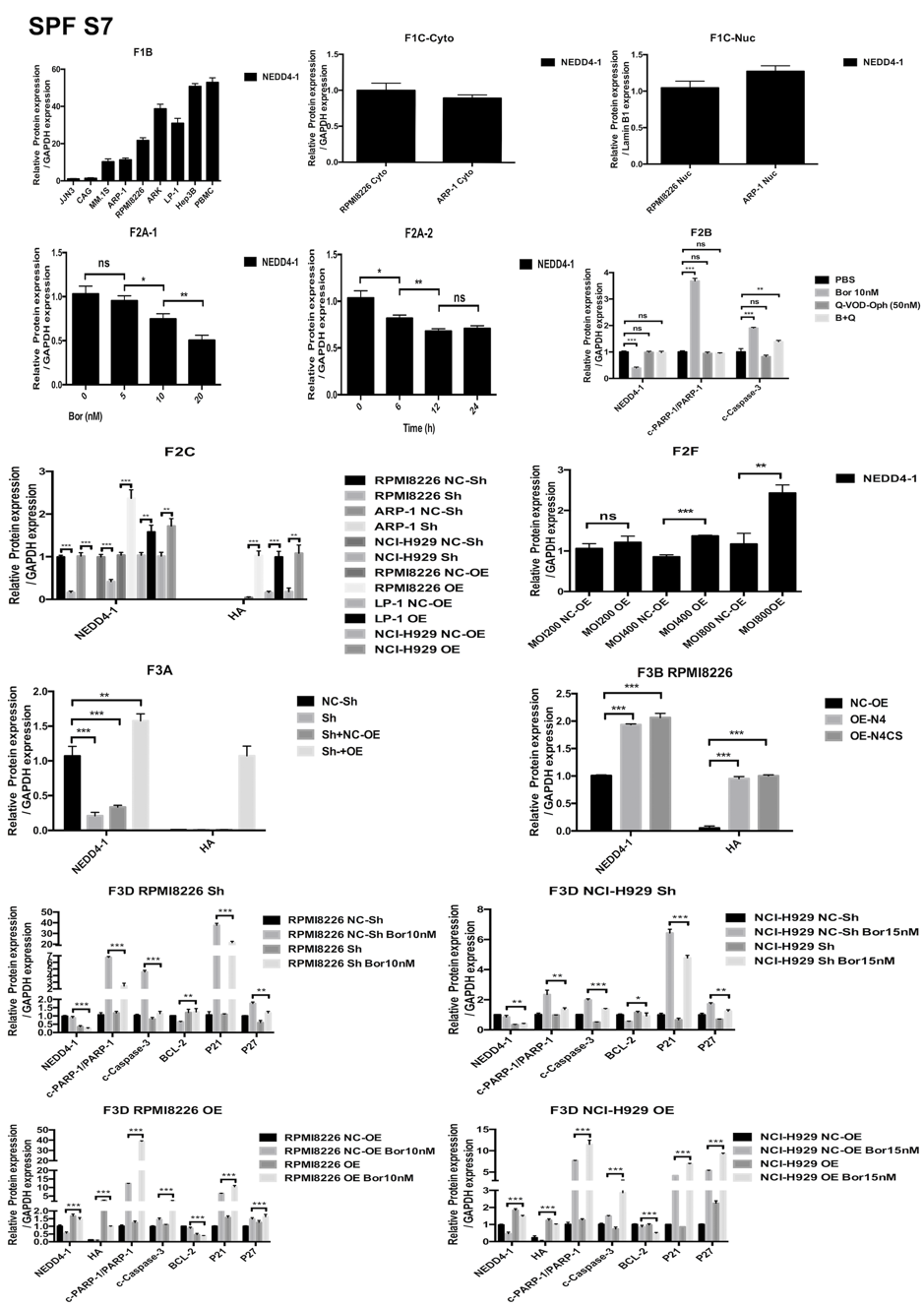


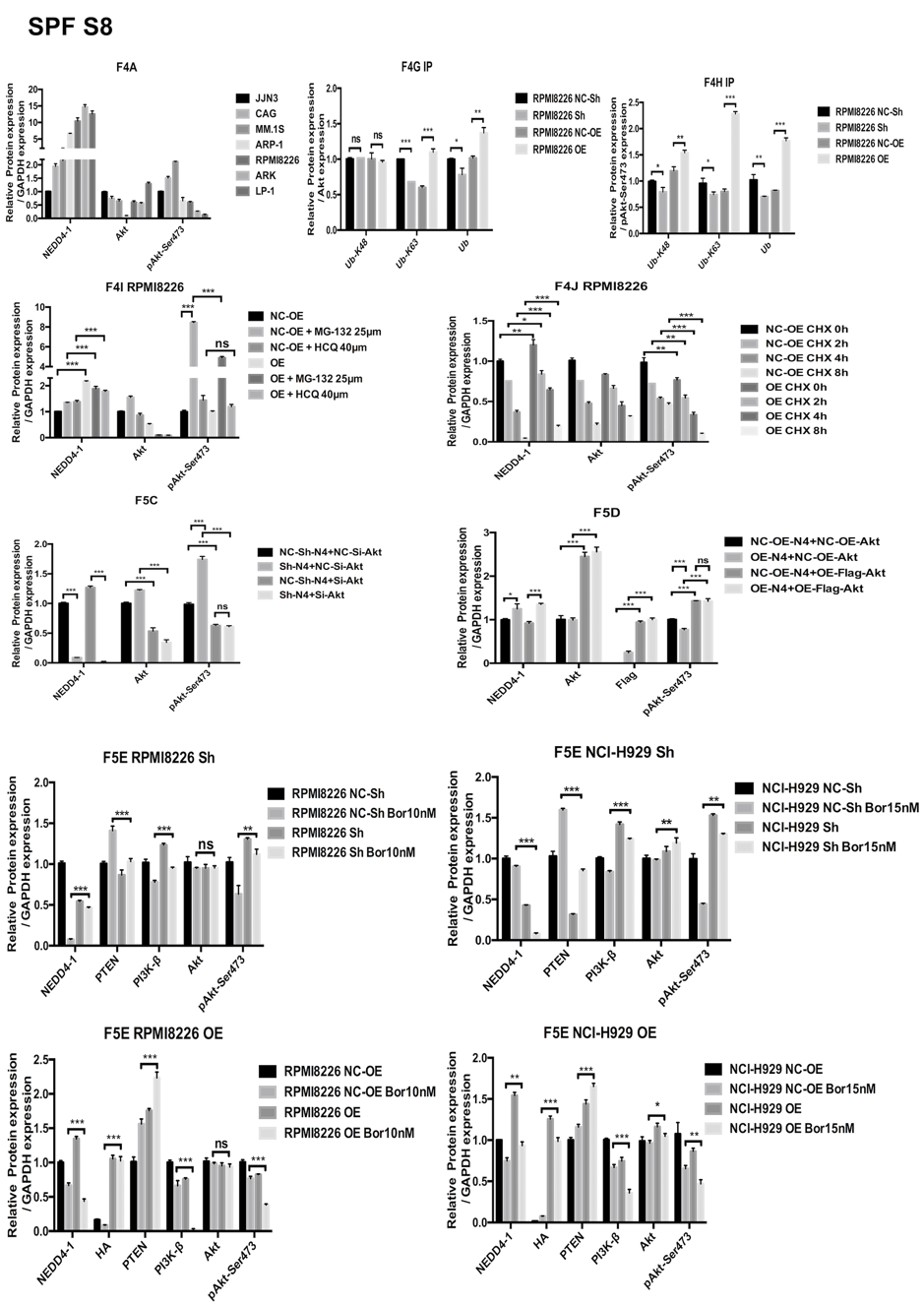


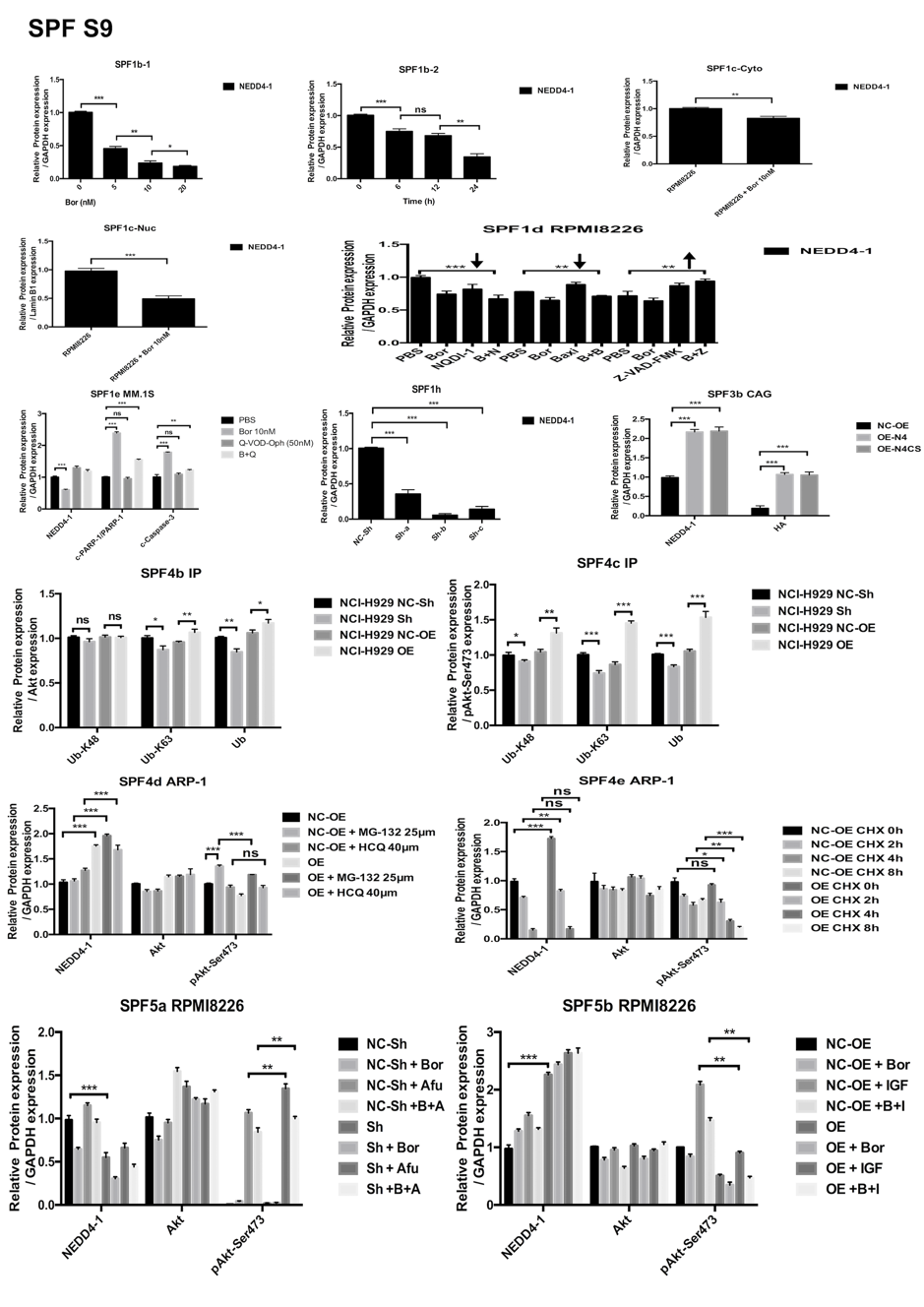

Supplement: Supplementary file 1 — Appendix S1: Supporting Information [file IJC-146-1963-s001.doc]
